# Differentially Expressed Hepatic Genes Revealed by Transcriptomics in Pigs with Different Liver Lipid Contents

**DOI:** 10.1155/2022/2315575

**Published:** 2022-01-28

**Authors:** Wentao Lyu, Yun Xiang, Xingxin Wang, Jingshang Li, Caimei Yang, Hua Yang, Yingping Xiao

**Affiliations:** ^1^State Key Laboratory for Managing Biotic and Chemical Threats to the Quality and Safety of Agro-Products, Institute of Agro-Product Safety and Nutrition, Zhejiang Academy of Agricultural Sciences, Hangzhou 310021, China; ^2^College of Animal Sciences & Technology, Zhejiang A & F University, Hangzhou 311300, China; ^3^Institute of Animal Husbandry and Veterinary Medicine, Jinhua Academy of Agricultural Sciences, Jinhua 321000, China

## Abstract

The liver is the center for uptake, synthesis, packaging, and secretion of lipids and lipoproteins. The research on lipid metabolism in pigs is limited. The objective of the present study is to identify the genes related to lipid metabolism and oxidative stress in pigs by using transcriptomic analysis. Liver segments were collected from 60 Jinhua pigs for the determination of liver lipid content. The 7 pigs with the highest and lowest liver lipid content were set as group H and group L, respectively. Liver segments and serum samples were collected from each pig of the H and L groups for RNA sequencing and the determination of triglycerides (TG) content and high-density lipoprotein cholesterol (HDL) content, respectively. The HDL content in the serum of pigs in the H group was significantly higher than the L group (*P* < 0.05). From transcriptomic sequencing, 6162 differentially expressed genes (DEGs) were identified, among which 2962 were upregulated and 3200 downregulated genes with the increase in the liver content of Jinhua pigs. After GO enrichment and KEGG analyses, lipid modification, cellular lipid metabolic process, cholesterol biosynthetic process, fatty acid metabolic process, oxidoreduction coenzyme metabolic process, oxidoreductase activity, acting on CH-OH group of donors, response to oxidative stress, nonalcoholic fatty liver disease (NAFLD), sphingolipid metabolism, and oxidative phosphorylation pathways were involved in lipid metabolism and oxidative stress in Jinhua pigs. For further validation, we selected 10 DEGs including 7 upregulated genes (*APOE*, *APOA1*, *APOC3*, *LCAT*, *CYP2E1*, *GPX1*, and *ROMO1*) and 4 downregulated genes (*PPARA*, *PPARGC1A*, and *TXNIP*) for RT-qPCR verification. To validate these results in other pig species, we analyzed these 10 DEGs in the liver of Duroc×Landrace×Yorkshire pigs. Similar expression patterns of these 10 DEGs were observed. These data would provide an insight to understand the gene functions regulating lipid metabolism and oxidative stress and would potentially provide theoretical basis for the development of strategies to modulate lipid metabolism and even control human diabetes and obesity by gene regulations.

## 1. Introduction

The liver is an essential metabolic organ and the central link for the carbohydrate, lipid, and protein metabolism [1]. The liver plays a unique role in controlling the glucose metabolism by maintaining the glucose concentration within the normal range. This is achieved through a strictly regulated enzyme and kinase system. These enzymes and kinases regulate the decomposition or synthesis of glucose in liver cells. The liver is the main processor of protein and amino acid metabolism, because it is responsible for most of the proteins secreted in the blood (whether based on the quality or range of unique proteins), the processing of amino acids for energy, and disposal of nitrogenous waste from protein degradation in the form of the urea metabolism. Moreover, the liver can also secrete bile for the digestion and decomposition of lipids and the absorption of fat-soluble vitamins [2]. The liver not only oxidizes lipids but also encapsulates the excess lipids, secretes them in other tissues, and stores them, such as adipose tissue. For lipid metabolism, the liver is the center for uptake, synthesis, packaging, and secretion of lipids and lipoproteins [3]. Hepatocytes are able to extract fatty acids from chylomicron remnants by lipoprotein lipase and oxidize fatty acids to provide energy for themselves and other organs [4]. For carbohydrate metabolism, the liver is capable to store, synthesize, metabolize, and release glucose [3]. Hepatic Krebs cycle allows the liver to maintain a high rate of biodegradation of carbohydrates and lipids to provide energy for the body [5, 6]. For protein and amino acid metabolisms, the liver could also synthesize, secrete, utilize, and metabolize proteins or amino acids [3]. Additionally, the liver could synthesize and secrete various lipoproteins after assembling fatty acids and glycerol into triglycerides. Therefore, the liver is a key connection between the lipid metabolism and glucose and protein metabolisms. Especially for pigs, the liver is one of the most important organs in regulating appetite and body weight as well as several metabolic processes [7].

Oxidative stress is caused by a sharp increase in free radicals in the body or a decline in the ability to scavenge free radicals, thereby disrupting the antioxidant-oxidation balance [8]. There are reports that oxidative stress is related to many diseases and is closely related to the health of the body [9, 10]. The free radicals that play a major role in oxidative stress are reactive oxygen species (ROS) and reactive nitrogen free radicals (RNS) [11]. Under physiological conditions, adipokines can induce the production of ROS and then produce oxidative stress, which in turn leads to the further deposition of lipid [12]. Therefore, understanding the relationship between lipid deposition and oxidative stress can reduce oxidative stress and promote body health by regulating lipid metabolism.

Transcriptome sequencing, also known as RNA sequencing (RNA-seq), is a technique to quantitatively describe the differences in gene types and expression levels at a global level [13]. RNA-seq is a powerful tool that can identify genes related to lipid metabolism and oxidative stress in the liver. Genes related to lipid metabolism were identified by using transcriptome analysis of liver samples in chickens and pigs [14, 15] to understand the effect of liver metabolism on lipid-related phenotypes. Regardless of whether it is for pigs or for humans, excessive lipid deposits will lead to obesity, which will cause a series of inflammatory reactions and eventually lead to a series of diseases, such as insulin resistance and diabetes [16].

As one of the most important economic animals, pigs are raised all over the world. Different breeds of pigs have significantly different genetic composition, which affects different physiological characteristics. The Jinhua pig, named after the city of Jinhua in East China's Zhejiang Province, is a traditional, slow-growing pig breed with a high body lipid content, early sexual maturity, and low fertility [17]. In the production of pigs, lipid deposition not only affects the growth efficiency of pigs but also affects the quality of pork. Excessive lipid deposition will reduce the lean meat rate of pigs, reduce the economic benefits of the pig industry, and also affect the flavor and quality of pork. Therefore, an in-depth understanding of the mechanism of pig lipid deposition can provide scientific targets for the rational regulation of pig lipid deposition. It has been approved that pigs are very similar to humans in terms of eating style, pancreatic shape and development level, gastrointestinal tract structure, metabolic level, and blood glucose level [18–20]. For phenotypic similarities of pigs to humans, it includes cardiovascular anatomy and function, metabolism, lipoprotein profile, size, tendency to obesity, and omnivorous habits. However, the current research on lipid metabolism and oxidative stress of Jinhua pigs is limited. Therefore, in this paper, liver samples were collected from 14 Jinhua pigs for transcriptome analysis to identify differential expressed genes related to lipid metabolism and oxidative stress. In this study, all pigs were from the same breeding line and fed under the same conditions, so we supposed all of the pigs in the experiment had similar average daily intake, allowing us to focus on differentially expressed genes that regulate lipid metabolism without being affected by the internal environment. It would provide a theoretical basis for the in-depth study of genes regulating lipid metabolism and oxidative in pigs and even for humans to develop strategies to modulate lipid metabolism and regulate related diseases caused by obesity.

## 2. Materials and Methods

### 2.1. Ethics Statement

All animal procedures were approved by the Institutional Animal Care and Use Committee of the Zhejiang Academy of Agricultural Sciences (ZAASDLSY2019-1910), and all methods were performed in accordance with the relevant guidelines and regulations.

### 2.2. Animals and Sampling of Animal Trial 1 for the Test

A total of sixty Jinhua castrated boars at 30 days old were raised in a commercial farm in Jinhua City, Zhejiang Province, China. All of the Jinhua pigs in this study were from the same breeding line, pedigreed Jinhua pigs, which is a Chinese local breed. Pigs were raised in pens with ad libitum access to diets and water. The diet was a commercial corn-soybean-based diet formulated with trace minerals and vitamins to meet the requirements of the National Research Council (NRC, 2012). After fasting for 12 h, all pigs were slaughtered at the age of 270 days. The average thickness of the backfat was measured on the first rib, last rib, and last lumbar vertebrae in the midline using a sliding caliper [18]. The liver segments were collected and stored at -80°C until RNA isolation and subsequent analysis.

### 2.3. Animals and Sampling of Animal Trial 2 for the Validation

In order to verify the results from animal trial 1, a total of 82 Duroc×Landrace×Yorkshire (DLY) pigs at 30 days old were fed with commercial corn-soybean-based diet under standardized environment. The commercial corn-soybean-based diet was formulated with to meet the requirements of the National Research Council (NRC, 2012). At the age of 180 days, all of pigs were sacrificed to measure backfat thickness and collect segments of liver for the determination of lipid content and lipid-related gene expression.

### 2.4. Determination of Liver Lipid Content

We used the Soxhlet [21] extraction method to determine the lipid content of the liver. We use an organic solvent to extract the lipid in the sample with Soxhlet extractor, make it dissolve in the organic solvent, and then evaporate the solvent, weigh the residual, and measure the lipid content in the sample. The liver lipid content was expressed as percentage of wet weight.

### 2.5. Histological Staining

Histological staining was performed as previously described with minor modifications [17]. Briefly, liver segments of Jinhua pigs were fixed in 4% paraformaldehyde for 1 h at room temperature, cryoprotected in 20% sucrose at 4°C overnight, and embedded in OCT. A series of 12 mm cryosections were prepared and stained with hematoxylin, eosin, and/or Oil Red O (Sigma-Aldrich, St. Louis, MO, United States). The liver sections were photographed by a light microscope (Nikon Corp., Tokyo, Japan).

### 2.6. Detection of Serum Biochemical Index

We determined serum biochemical parameters, including triglycerides (TG) and high-density lipoprotein cholesterol (HDL), using conventional enzymatic determination kits (Nanjing Jiancheng Institute of Bioengineering, Nanjing, Jiangsu, China) and an automatic biochemical analyzer (Hitachi, Tokyo, Japan).

### 2.7. RNA Extraction

Total RNA from liver samples was extracted using TRIzol reagent (Invitrogen, Carlsbad, CA, USA) strictly according to the manufacturer's instructions. A NanoPhotometer® spectrophotometer (IMPLEN, MD, CA, USA) was used to detect RNA purity (OD260/280 and OD260/230 ratios), and an Agilent 2100 Bioanalyzer (Agilent Technologies, Santa Clara, CA, USA) was used to accurately detect RNA integrity.

### 2.8. Library Preparation and Transcriptome Sequencing

A total amount of 1 *μ*g of RNA per sample was used as the input material for RNA sample preparation. Sequencing libraries were generated using the NEBNext® Ultra™ RNA Library Prep Kit for Illumina® (NEB, Ispawich, CA, USA) following the manufacturer's recommendations, and index codes were added to attribute sequences to each sample. To preferentially select cDNA fragments that were 250~300 bp in length, the library fragments were purified with the AMPure XP system (Beckman Coulter, Beverly, USA). Finally, PCR products were purified (AMPure XP system) (Beckmankurt life sciences division, Indianapolis, Indiana, USA), and library quality was assessed on the Agilent Bioanalyzer 2100 system.

For RNA-seq, a 2 × 150 bp paired-end sequencing was performed in the present study. Clustering of the index-coded samples was performed on a cBot Cluster Generation System using TruSeq PE Cluster Kit v3-cBot-HS (Illumina) according to the manufacturer's instructions. After cluster generation, the library preparations were sequenced on an Illumina NovaSeq 6000 platform, and paired-end reads were generated. The raw transcriptome read data are available in the SRA database under accession number PRJNA721126.

### 2.9. Data Processing

Raw reads of fastq format were firstly processed through in-house Perl scripts to remove adapter sequences [22], ploy-N sequences, and low-quality reads. Quality parameters of Q20, GC content, and sequence duplication level were used for further data filtration. All the downstream analyses were based on the clean reads. The reference genome and gene model annotation files were downloaded from genome website directly (https://www.ncbi.nlm.nih.gov/genome/?term=Sus+scrofa). Hisat2 v2.0.5 was used to build the index of the reference genome and align the paired-end clean reads to the reference genome [23].

### 2.10. Differential Expression Analysis

Read counts were generated for each gene using featureCounts v1.5.0-p3 [24]. The expression level for each gene was normalized to quantify fragments per kilobase of transcript sequence per million base-pairs sequenced (FPKM) [25, 26]. The differential expression analysis of two groups was performed using the DESeq2 R package (v1.16.1) [27]. Gene-based expression matrix (with default normalization) was used to compare boars from the H and L groups. DESeq2 provides statistical routines for determining differential expression in digital gene expression data using a model based on the negative binomial distribution. The resulting *P* values were adjusted using the Benjamini and Hochberg approach for controlling the false discovery rate [28]. Genes with an adjusted *P*adj < 0.05 and ∣log2fold change | >1 found by DESeq2 were assigned as differentially expressed.

The clusterProfiler R package was used to implement Gene Ontology (GO, http://www.geneontology.org/) enrichment analysis of the differentially expressed genes (DEGs) and test the statistical enrichment of DEGs in Kyoto Encyclopedia of Genes and Genomes (KEGG, http://www.genome.jp/kegg/) pathways [29]. GO terms and KEGG pathways with corrected *P* value less than 0.05 were considered significantly enriched. GO terms and KEGG pathways were annotated with InterProScan (http://www.ebi.ac.uk/Tools/pfa/iprscan/) [30] and KOBAS (3.0.3) [31], respectively.

### 2.11. Validation of Differentially Expressed Genes by RT-qPCR

To demonstrate the repeatability and precision of the RNA-seq gene expression data derived from the liver tissue libraries, a CFX384 multiple real-time fluorescence quantitative PCR instrument was used for analysis. The real-time quantitative PCR (RT-qPCR) system (20 *μ*L) was as follows: power SYBR® Green Master Mix, 10 *μ*L; upstream and downstream primers (10 *μ*mol/L), 0.5 *μ*L; sterilized distilled water, 8 *μ*L; and cDNA template, 1 *μ*L. The reaction conditions were as follows: 95°C for 1 min, followed by 40 cycles of 95°C for 15 sec and 63°C for 25 sec (for collecting fluorescence data). Finally, the melting curve was drawn at 55-95°C. The reaction for each sample was repeated three times, and the relative expression level of each gene was statistically analyzed as 2 (Ct internal reference gene-Ct target gene). The primers used for quantification in the study were designed using Primer-BLAST on the NCBI website (https://www.ncbi.nlm.nih.gov/tools/primer-blast/). The gene information for real-time PCR is shown in Table 1, with GAPDH serving as the internal reference gene [32].

### 2.12. Statistical Analysis

Data are expressed as the mean ± standard error of mean (SEM). All statistical analyses were performed in SPSS version 23. All figures are generated in GraphPad Prism V8.0 and OriginLab 2018. The difference in liver lipid content and backfat thickness between two groups was analyzed by unpaired two-tailed Student's *t*-test and considered significant when the *P* value was no more than 0.05.

## 3. Results

### 3.1. Lipid-Related Phenotypes of Jinhua Pigs

**Figure 1:**
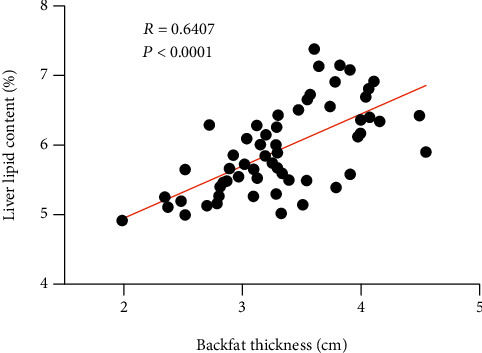
The linear regression analysis between the backfat thickness and liver lipid content. The round black dots indicate the 60 Jinhua pigs.

To study the relationship between backfat thickness and liver lipid, we conducted a correlation analysis of backfat thickness. The average backfat thickness and average liver lipid content were 3.347 ± 0.5567 cm and 5.976 ± 0.664%, respectively (Figure 1). The linear regression revealed a positive correlation between backfat thickness and liver lipid content (*R* = 0.6407, *P* < 0.001).

**Figure 2:**
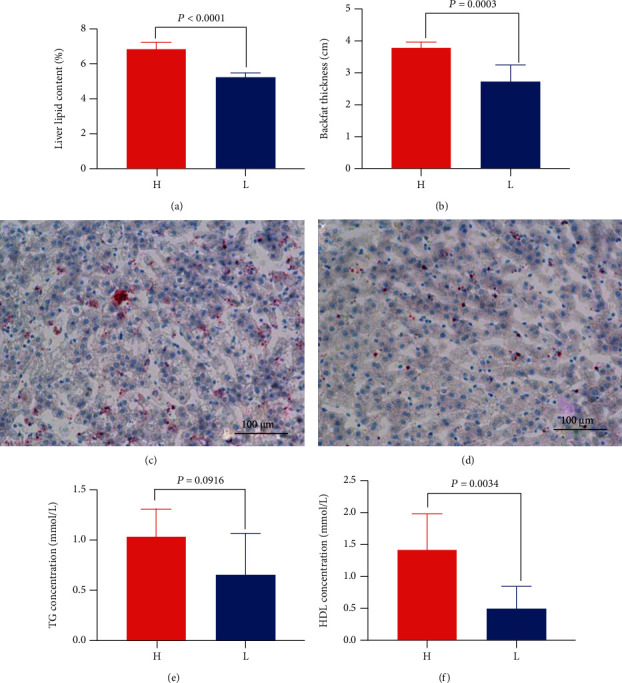
Lipid-related phenotypes of Jinhua pigs in group H and group L. (a) The liver lipid content of different groups (*n* = 7). (b) The thickness of backfat in different groups (*n* = 7). (c) Liver sections were enlarged to 100 *μ*m in group H.(d) Liver sections were enlarged to 100 *μ*m in group L. (e) Serum TG concentration in different groups (*n* = 7). (f) Serum HDL concentration in different groups (*n* = 7). H: the high liver lipid content group; L: the low liver lipid content group. Red dots in (c) and (d) indicate lipid droplets. Data were expressed as mean ± SEM with statistical analysis by unpaired two-tailed Student's *t*-test.

For further investigation, we sorted these 60 Jinhua pigs from high to low according to the liver lipid content and set the highest 7 pigs and the lowest 7 pigs as group H and group L, respectively. The mean value of liver content in the H group was 7.19% while that in the L group was 5.08%, showing a significant difference (*P* < 0.0001, Figure 2(a)). As expected, the backfat thickness in group H was significantly higher than that in group L (*P* = 0.0003, Figure 2(b)).

To convince the liver lipid content, we performed the H.E. staining of liver segments. As shown in Figures 2(c) and 2(d), it was clear that liver lipid content in group H is higher than that in group L.

The average concentration of TG in the serum of group H was 1.033 mmol/L, the average concentration of group L was 0.649 mmol/L, and there was no difference, but group H was higher than group L (*P* = 0.0916, Figure 2(e)). The average concentration of HDL in the serum of group H was 1.591 mmol/L, the average concentration of group L was 0.5515 mmol/L, group H was higher than group L, and the difference was significant (*P* = 0.0034, Figure 2(f)).

### 3.2. Summary of RNA-seq Data

**Figure 3:**
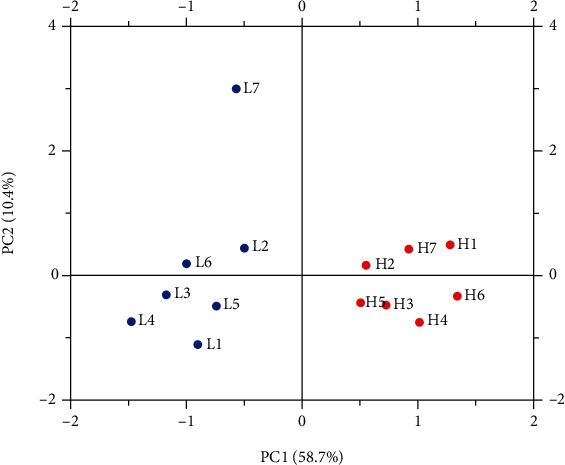
Differences between transcriptome replicates of the H and L groups' ducks based on the principal component analysis. Note: the abscissa is the first principal component, and the ordinate is the second principal component.

The average number of original reads for 14 samples was 46996754. After quality control of the original reads with Q20, sequence duplication level, and GC content, there was an average of 45809803 clean reads per sample, accounting for 97.48% of the original reads. The average Q30 value, which is the percentage of bases for which the recognition accuracy exceeds 99.9%, was 95.12%. The samples were of good quality, and the average number of clean bases was 6.87 GB, averaging over 6 GB. The percentage of reads aligned to the unique location of the reference genome was 95.73% to 96.89% among the clean reads, and the average mapping rate of clean reads mapped to reference genes was 96.41% (Table 2). In summary, the sequencing data was qualified for the subsequent data analysis. To assess intergroup differences and intragroup sample duplication, we performed PCA analysis on readcount of all samples, and the results showed intragroup aggregation and intergroup isolation (Figure 3).

### 3.3. Differentially Expressed Genes Analysis

**Figure 4:**
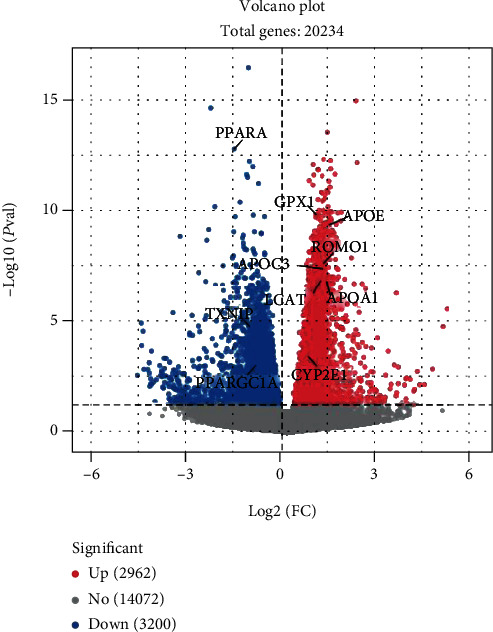
Volcano plot of the total expression of genes in both the H and L groups. A total of 21234 genes were expressed in both the H and L groups, and there were 6162 differentially expressed genes (DEGs). The *x*-axis represents the log2 fold change values for gene expression, and the *y*-axis represents the −log10 significance of the difference in the expression (*P*adj < 0.05). Red dots indicate 2962 upregulated DEGs, blue dots indicate 3200 downregulated DEGs, and gray dots indicate 14072 nondifferentially expressed genes.

**Figure 5:**
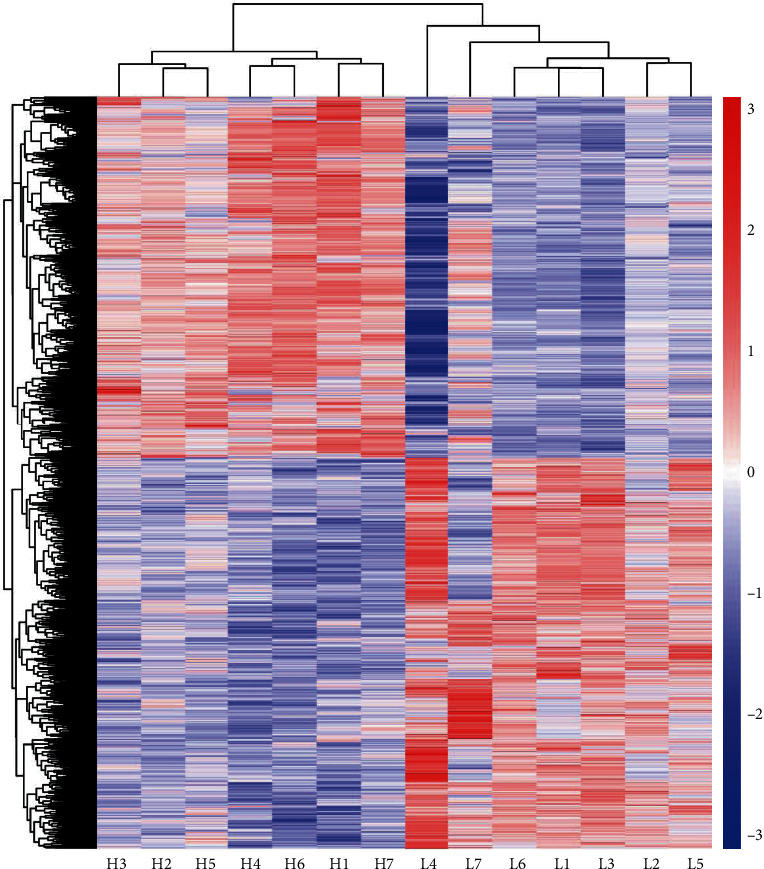
Liver tissue expression profiles of 6162 differentially expressed genes (DEGs) in the H vs. L groups. Hierarchical clustering analysis of *z*-scored FPKM was performed for each DEG between Jinhua pigs in the H and L groups. Colour scale represents FPKM normalized log10 transformed counts. Horizontal bars represent genes. The vertical column represents samples. Red colour indicates upregulated genes, while blue colour indicates downregulated genes.

Group H compared with group L, with *P*adj < 0.05 and ∣log − 2fold change | >0 as the threshold, a total of 6162 DEGs were identified. Among them, we identified 2962 upregulated genes and 3200 downregulated genes when comparing group H to group L (Figure 4). The DEG expression patterns of each sample were clustered on the basis of the log2 (fold change) values of their expression ratios, which exhibited good repeatability of samples in two groups (Figure 5).

### 3.4. GO Annotation and Enrichment Analysis of Differentially Expressed Genes

**Figure 6:**
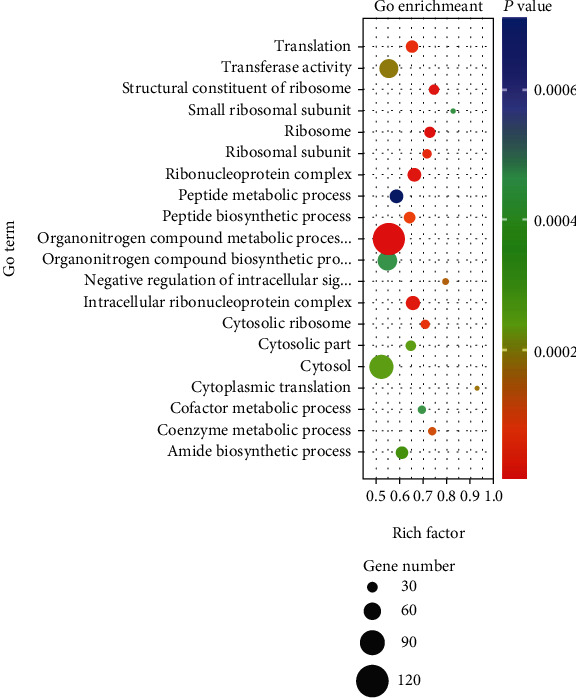
GO term enrichment analysis of differentially expressed genes (DEGs). The 20 top terms were obtained by GO enrichment, including 10 terms for biological process, 8 term for cell component, and 2 terms for molecular function. The size of the dots indicates the number of expressed genes in the pathways, and the colour of the dots represents the *P* value of the significant pathway.

To further elucidate the functional roles of the 6162 DEGs, GO term enrichment analysis was performed to search for significantly overrepresented categories. A total of 178 terms (Table S1) were significantly enriched in the three categories (*P* < 0.05), including biological process, cellular component, and molecular function. The top 20 terms (the 20 with the lowest *P* value) which include 10 terms for biological process, 8 terms for cell component, and 2 terms for molecular function were further analyzed to determine the associated regulatory functions (Figure 6). Four terms (Table 3) were related to lipid metabolism, namely, lipid modification (GO:0030258), cellular lipid metabolic process (GO:0044255), cholesterol biosynthetic process (GO:0006695), and fatty acid metabolic process (GO:0006641). And three terms (Table 4) were related to oxidative stress, oxidoreduction coenzyme metabolic process (GO:0006733), oxidoreductase activity, acting on CH-OH group of donors (GO:0016614), and response to oxidative stress (GO:0006979). In addition, there were some genes involved in lipid metabolism and oxidative stress in these terms, such as *APOE*, *APOA1*, *APOC3*, *LCAT*, *CYP2E1*, *PPARGC1A*, *GPX1*, *ROMO1*, and *TXNIP*.

### 3.5. KEGG Pathway Analysis of DEGs

**Figure 7:**
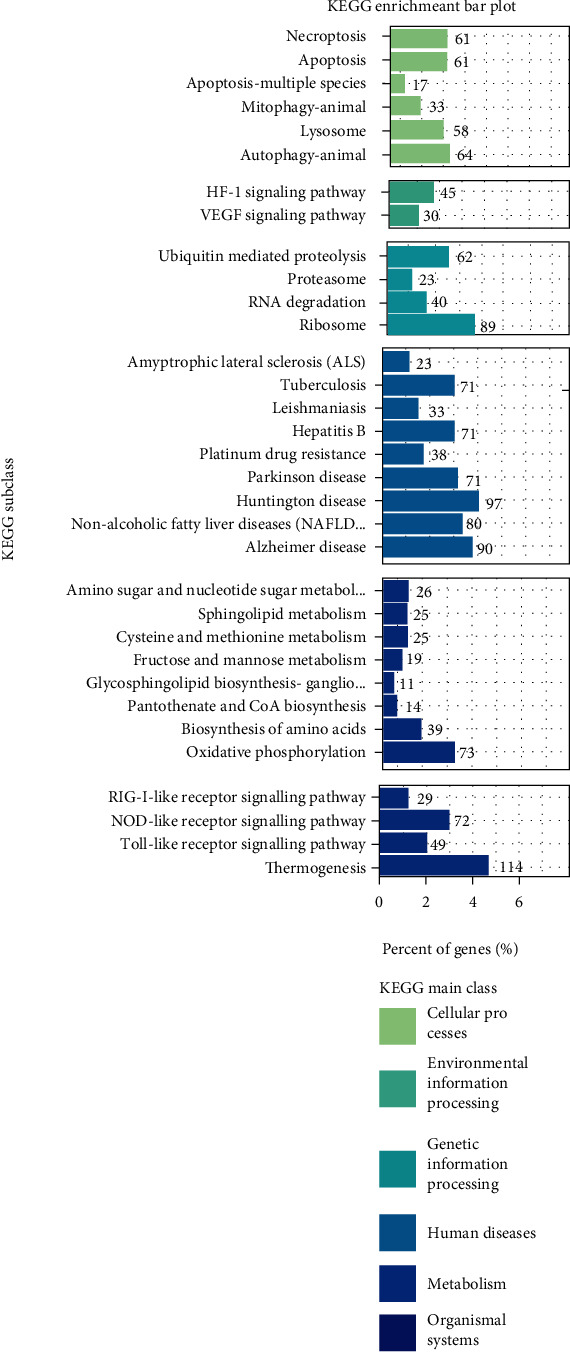
KEGG pathway enrichment analysis of differentially expressed genes (DEGs).

To identify the pathways those DEGs involved, we integrated the 6162 DEGs into the KEGG pathway database, and a total of 33 pathways (*P* < 0.05) were significantly enriched (Figure 7, Table S2). There were 2 pathways involved in lipid metabolism (Table 5), including nonalcoholic fatty liver disease (NAFLD) (ssc04932) and sphingolipid metabolism (ssc00600). And oxidative phosphorylation (ssc00190) was related to oxidative stress (Table 5). There were 10 significantly enriched genes shown in Table 6 which were related to lipid metabolism and oxidative stress. Among them, 4 genes, *APOE*, *PPARGC1A*, *CYP2E1*, and *TXNIP*, were highly enriched both in GO terms and significantly expressed in KEGG pathways; 5 genes, *APOA1*, *APOC3*, *LCAT*, *GPX1*, and *ROMO1*, were only enriched in GO terms; and *PPARA* was only enriched in KEGG pathways.

### 3.6. DEG Expression in the Liver of Jinhua Pigs

**Figure 8:**
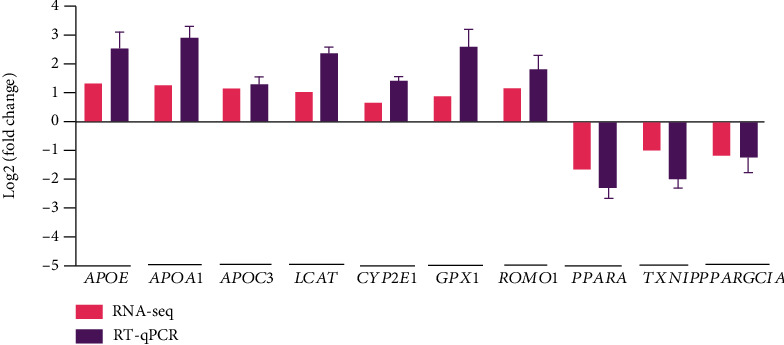
Validation of 10 DEGs by RT-qPCR in the liver of Jinhua pigs. The log2(fold change) was calculated when each indicated DEGs in the H group was compared to the L group. For the results of RT-qPCR, the 2^−ΔΔCt^ method was used to determine the relative expression level of each indicated DEGs in the H group over the L group.

To validate the RNA-seq results, 10 DEGs, including 7 upregulated genes (*APOE*, *APOA1*, *APOC3*, *LCAT*, *CYP2E1*, *GPX1*, and ROMO1) and 3 downregulated genes (*PPARA*, *PPARGC1A*, and *TXNIP*), were selected for RT-qPCR analysis in the liver of Jinhua pigs. As expected, all the selected DEGs showed a concordant expression pattern between the RNA-seq and qPCR results (Figure 8).

### 3.7. Validation of the Lipid-Related Phenotypes and Gene Expression in DLY Pigs

**Figure 9:**
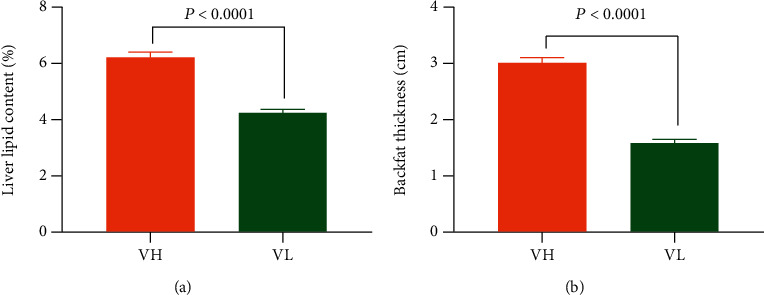
The liver lipid content and backfat thickness of DLY pigs in the VH and VL groups. (a) The liver lipid content of different groups (*n* = 7). (b) The thickness of backfat in different groups (*n* = 7). Data was expressed as mean ± SEM with statistical analysis by unpaired two-tailed Student's *t*-test. VH: validation high group including pigs with high liver lipid content; VL: validation low group including pigs with low liver lipid content.

In order to validate the results from trial 1, we determined the liver lipid content of 82 DLY pigs, and we set the 7 pigs with the highest liver lipid content as the validation high (VH) group and the 7 pigs with the lowest liver lipid content as the validation low (VL) group. The mean of liver lipid content in the VH group was 6.24% while that in VL group was 4.26%, showing a significant difference (*P* < 0.0001, Figure 9(a)). As expected, the backfat thickness in the VH group was significantly higher than that in the VL group (*P* < 0.0001, Figure 9(b)).

**Figure 10:**
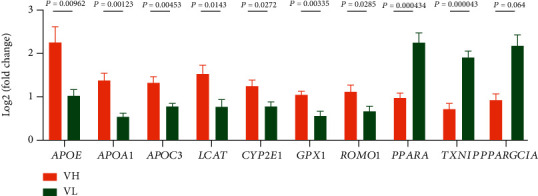
Validation of 10 related genes by RT-qPCR in the liver of DLY pigs. For the results of RT-qPCR, the 2^−ΔΔCt^ method was used to determine the relative expression level of 10 related genes in the VH group over the VL group. VH: validation high group including pigs with high liver lipid content; VL: validation low group including pigs with low liver lipid content.

In order to verify the reproducibility of the above results on the other breeds of pigs, RT-qPCR verification of 10 lipid-related genes, namely, *APOE*, *APOA1*, *APOC3*, *LCAT*, *CYP2E1*, *GPX1*, *ROMO1*, *PPARA*, and *PPARGC1A*, were performed on the liver tissues of the DLY pigs in the VH and VL groups. It was found that the expression patterns of these 10 genes were consistent with those in Jinhua pigs (Figure 10) with different liver lipid contents.

## 4. Discussion

As one of the most popular local pig breed in China, Jinhua pigs are famous for the superior meat quality with a higher body lipid content than the commercial pig breeds such as Landrace, Yorkshire, and Duroc [33]. The liver plays an important role in lipid metabolism, which is capable to secrete bile and bile acid salt emulsifying lipids to promote the digestion and absorption of lipids [4]. However, the data on lipid metabolism in Jinhua pigs is limited. In this study, transcriptome analysis was performed in the 14 liver samples to investigate the possible regulation of lipid deposition in Jinhua pigs. Similarly, Wang et al. [34] used RNA-seq technology to study the lipid metabolism mechanism in the liver of Yorkshire pigs and Anqing six-end-white pigs. Several genes responsible for lipid metabolism have been identified including *PPARA*, *PCK1*, *CYP7A1*, *PLIN1*, *ACSL3*, and *RetSat*. Xing et al. [35] conducted RNA-seq analysis on the livers of Songliao black pigs with high and low backfat thickness and identified genes involved in lipid regulation which played an important role in liver lipid and fatty acid metabolism, such as *FABP1*, *LCN2*, *PLIN2*, *CYP1A1*, *CYP1A2*, *CYP2A6*, and *CYP26A1*. Obesity is caused by excessive accumulation of lipid and is considered to be the main potential factor for the onset of many diseases (diabetes, cardiovascular, liver diseases, etc.), and these symptoms are all related to oxidative stress [12]. And oxidative stress is one of the factors restricting the pig industry, which has a certain impact on the production performance and health of pigs [8]. We used a larger pig population with 60 individuals and analyzed 14 liver samples, making our results more typical. In order to verify the repeatability of Jinhua pig lipid-related phenotypes and gene expression in other pig species, we conducted RT-qPCR to verify the expression levels of related genes in DLY white pigs, and the results showed that the expression patterns of related genes in DLY pigs were consistent with those in Jinhua pigs. This shows that our study is universal.

Apolipoproteins is a part of plasma lipoprotein, which is mainly divided into five categories: apolipoproteins A, B, C, D, and E. The basic function of apolipoproteins is to carry lipids and stabilize the structure of lipoproteins. Some apolipoproteins also have functions such as activating lipoprotein metabolism enzymes and recognizing receptors. It plays an important role in lipid transportation and metabolism. Additionally, apolipoproteins are the main component of very low-density lipoprotein (VLDL), chylomicrons (CM), and HDL [36]. Apolipoprotein A1 (*APOA1*), apolipoprotein C3 (*APOC3*), and apolipoprotein E (*APOE*) were positively regulated lipid deposition in the liver of Jinhua pigs. Studies have shown that *APOE* could also mobilize cholesterol in cells and tissues. A special class of amphiphilic apolipoproteins, like *APOA1* and *APOE*, could combine with ATP to interact with ATP-binding cassette subfamily A member 1 (*ABCA1*), forming a discoidal complex of phospholipids and apolipoproteins [37–39]. The role of this discoidal complex is to dissolve excess cholesterol existing in the plasma membrane of the cell or cholesterol shed from the cell to the extracellular matrix [40, 41]. Lecithin cholesterol acyltransferase (*LCAT*) can enhance the ability of this discoidal complex to dissolve cholesterol [42]. LCAT is an enzyme secreted by the liver that can esterify cholesterol in the center of HDL. With the actions of *APOA1*, *APOD*, and *APOE*, it would result in the further increase in the volume of HDL and promote lipid deposition [43]. *APOC3* was related to the regulation of LPL activity. Studies have found that the overexpression of *APOC3* will inhibit the activity of *LPL*, thereby increasing the triglyceride content, leading to lipid deposition [44]. Therefore, it is not surprising to find *APOE*, *APOA1*, *APOC3*, and *LCAT* giving a higher expression in the liver of the pigs in the H group than that in the L group.

Peroxisome proliferator-activated receptor alpha (*PPARA*) and PPARG coactivator 1 alpha (*PPARC1A*) belong to peroxisome proliferator-activated receptors (PPARs) family. Among them, PPARA is mainly expressed in liver cells, cardiomyocytes, and brown adipocytes. PPARA participates in the metabolism of sphingolipids in the liver. Sphingolipids are a class of complex lipids with a ceramide structure, which are essential for various biological processes including development and growth [45]. *PPARA* is not only involved in lipid metabolism in the liver but also related to the *β*-oxidation of mitochondria. Studies have shown that the constitutive mitochondrial *β*-oxidation activity in the liver of PPARA knockout mice is significantly reduced [46]. *PPARGC1A* was first discovered and reported in the screening of mouse brown lipid cDNA library [47]. As a key nuclear transcription coactivator, *PPAGC1A* can bind to many different transcription factors, participate in a series of orderly metabolic processes, and play an important role in regulating mitochondrial biosynthesis, sugar metabolism, fatty acid oxidation, and muscle fiber type conversion [48–50]. *PPARGC1A* also plays a key role in regulating the redox environment of cells by upregulating the functions of antioxidant genes and their derivatives [51] and interacts with PPARs to increase fatty acid oxidation (FAO) [52]. Studies have shown that lysosomes can inhibit the expression of *PPARA* by inhibiting the expression of *PPARGC1A*, thereby causing lipid accumulation in the liver [53]. In this experiment, the expression levels of *PPARA* and *PPARGC1A* were both downregulated, and the lysosomal pathway was enriched and expressed in the KEGG pathway, so this can regulate lipid metabolism in the liver through the above process.

Among the members of the cytochrome P-450 family, the cytochrome P450 family 2 subfamily E member 1 (*CYP2E1*) has been extensively studied because it is metabolically activated by a variety of xenobiotics and carcinogens (including nitrosamines, benzene, vinyl chloride, and halogenated solvents). It is a key enzyme and has a significant contribution to the metabolism of ethanol to acetaldehyde [54]. In the liver, *CYP2E1* is mainly expressed in the endoplasmic reticulum of hepatocytes in the lobular center, which has high NADPH oxidase activity, that can lead to the production of ROS and significantly promote the induction of oxidative stress in many pathological conditions [55]. *CYP2E1* is related to oxidative stress. In this experiment, *CYP2E1* was upregulated in the H group. One of the most important proteins in the inner mitochondrial membrane is ROS modulator 1 (*ROMO1*), which interferes with the production of ROS, and as the rate of this protein increases, oxidative stress increases, which ultimately leads to some diseases [56]. Studies have shown that the increase in Romo1 expression enhances cellular ROS levels and oxidative DNA damage [57], which consistent with our experimental results. The thioredoxin-interacting protein (*TXNIP*) is a multifunctional adaptor protein for different signaling pathways. *TXNIP* is a multifunctional adaptor protein for different signaling pathways [58]. The main role of *TXNIP* is to negatively regulate the function of thioredoxin (TRX) by inhibiting its reducing ability and promoting cellular oxidative stress [59]. Studies have shown that high levels of *TXNIP* inhibit the redox activity of cytoplasmic TRX1 and increase the level of ROS. On the contrary, ROS can negatively regulate the expression of *TXNIP*. *GPX1* is an important antioxidant enzyme involved in preventing the harmful accumulation of hydrogen peroxide in cells [60]. It is present in all cells and has been found to be more effective than catalase in removing intracellular peroxides under many physiological conditions [61]. *GPX1* can also reduce lipid hydroperoxides and other soluble hydroperoxides [62] and can reduce 1-linoleoyl lysophosphatidylcholine hydroperoxide, but not tri- or diacylglycerol hydroperoxides [63]. *GPX1* cannot be replaced by any other selenoproteins in protecting against systemic oxidative stress, and *GPX1* has the main antioxidant function in the body [64]. Studies have shown that overexpression of *GPX1* can protect mice under oxidative stress, but it can still cause obesity and diabetes [65].

**Figure 11:**
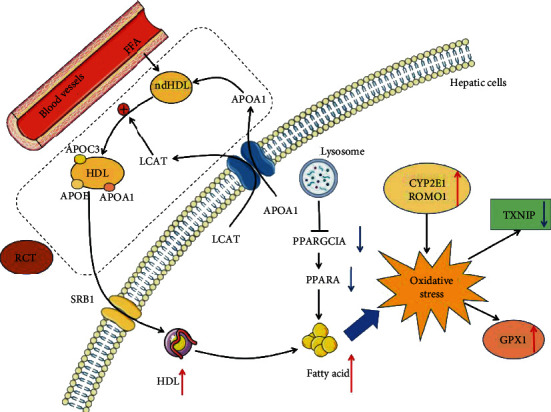
Overview of liver lipid metabolism and oxidative stress. RCT: reverse cholesterol transport.

The accumulation of lipid in the liver is mainly due to a problem in the balance between lipid acquisition and processing [66]. Based on the results above, we summarized the pathways of the lipid metabolism in the liver of Jinhua pigs (Figure 11). *APOA1* is produced and secreted from the liver and released into the plasma to combine with free fatty acids (FFA) to form a ndHDL. Once ndHDL is produced, it will cause cholesterol efflux, and then, ndHDL will absorb the efflux cholesterol; then, it will be esterified by *LCAT* to produce HDL, which carries with *APOA1*, *APOC3*, and *APOE*. It is then transported back to the liver by scavenger receptor B1 (*SRB1*), leading to lipid deposition in the liver. On the other hand, lysosomes could inhibit the expression of *PPARGC1A*, thereby reducing the expression of *PPARA* as well as resulting in lipid deposition in the liver. Excessive lipid deposition as well as overexpression of *CYP2E1* and *ROMO1* would promote oxidative stress, further inducing the expression of the antioxidant enzyme *GPX1* with high ROS levels suppressing the expression of *TXNIP*.

## 5. Conclusions

In this study, we analyzed the DEGs in pigs with different liver lipid contents. Through GO enrichment analysis and KEGG analysis, it is found that 10 DEGs, namely, *APOE*, *APOA1*, *APOC3*, *LCAT*, *PPARA*, *PPARGC1A*, *CYP2E1*, *GPX1*, *ROMO1*, and *TXNIP*, displayed a crucial regulatory role in lipid metabolism and oxidative stress in the liver of pigs. This study provided insights into the molecular mechanism for regulating lipid metabolism and oxidative stress in pigs even humans.

## Figures and Tables

**Table 1:** Primers for RT-qPCR.

Gene name	Primer sequences (5′ to 3′)	TM (°C)	Product size (bp)
GAPDH	F: CCAGGGCTGCTTTTAACTCTG	60	104
	R: GTGGGTGGAATCATACTGGAACAT		

APOE	F: GGTGCAGTCCCTGTCTGA	60	79
	R: CTCTATCAGCTCCGTCAGTTC		

APOC3	F: GACACCTCCCTTCTGGACAAA	60	86
	R: GACTCCTTCACGCTGGTTAG		

APOA1	F: GAAGGATTTTGCCACCGTGTATG	60	107
	R: GGAGTTTCAGGTTGAGGTGTTTTC		

LCAT	F: CGGCTGGAGCCCAGTTATATG	60	144
	R: CCCAGCAAGCTTCAGGTAGTA		

PPARA	F: GTTGCAAGGGCTTCTTTCGG	60	129
	R: CCGAGAGGCACTTGTGGAAA		

PPARGC1A	F: GCTTGACGAGCGTCATTCAG	60	100
	R: GGTCTTCACCAACCAGAGCA		

CYP2E1	F: CACAAGGACAAAGGGGTCATTT	60	110
	R: TGCTCATTGCCCTGTTTCCC		

GPX1	F: TCCAGTGTGTCGCAATGACA	60	102
	R: TCGATGGTCAGAAAGCGACG		

ROMO1	F: GCGTGAAGATGGGCTTTGTG	60	135
	R: TCTGCATCATGGTTTTCCCGA		

TXNIP	F: CATGTTCCCGCATTGTGGTG	60	100
	R: ACCGATGACAACTTCTGCGT		

**Table 2:** Summary of sequence quality and alignment information from the liver transcriptome analysis of Jinhua pigs.

Sample	Read numbers	Clean reads	Clean ratio (%)	Clean bases	Q30 (%)	Total mapped	Mapped ratio (%)
H1	46615168	45213222	96.99	6.85GB	95.29	43613957	96.46
H2	47224788	46062522	97.54	6.76GB	95.22	44406844	96.41
H3	46528606	45425370	97.63	6.72GB	95.36	43487077	95.73
H4	45712164	44501604	97.35	6.77GB	95.24	42959852	96.54
H5	46089942	45064114	97.77	6.88GB	95.33	43422246	96.36
H6	49504486	48370168	97.71	6.79GB	94.72	46645521	96.43
H7	47692912	46119656	96.70	7.24GB	95.43	44351571	96.17
L1	46532476	45643802	98.09	6.78GB	95.31	44141776	96.71
L2	46016438	45069046	97.94	6.91GB	95.45	43475540	96.46
L3	46299018	45354188	97.96	6.83GB	94.81	43944203	96.89
L4	46875078	45157476	96.34	6.68GB	95.03	43471601	96.27
L5	46972136	45833438	97.58	6.76GB	94.99	44268232	96.59
L6	46450896	45277662	97.47	7.26GB	95.16	43693079	96.50
L7	49440452	48244974	97.58	6.92GB	94.37	46387276	96.15

**Table 3:** The significantly enriched terms associated with lipid metabolism.

Term ID	Description	*P* value	Gene number
GO:0030258	Lipid modification	0.003820271	14
GO:0044255	Cellular lipid metabolic process	0.029944475	40
GO:0006695	Cholesterol biosynthetic process	0.033857412	8
GO:0006631	Fatty acid metabolic process	0.036584082	21

**Table 4:** The significantly enriched terms associated with oxidative stress.

Term ID	Description	*P* value	Gene number
GO:0006733	Oxidoreduction coenzyme metabolic process	0.014110964	13
GO:0016614	Oxidoreductase activity, acting on CH-OH group of donors	0.033328294	18
GO:0006979	Response to oxidative stress	0.038686219	19

**Table 5:** The significantly enriched pathways associated with lipid metabolism and oxidative stress.

Pathway ID	Description	*P* value	Gene number
ssc04932	Nonalcoholic fatty liver disease (NAFLD)	1.55503*E* − 05	49
ssc00600	Sphingolipid metabolism	0.021170698	25
ssc00190	Oxidative phosphorylation	2.1411*E* − 05	73

**Table 6:** Information of 16 DEGs associated with lipid metabolism and oxidative stress.

Gene name	Gene ID	log2FoldChange	*P* value	Descriptions
*APOE*	397576	1.34	4.58*E* − 10	Apolipoprotein E
*APOA1*	397691	1.26	1.69*E* − 07	Apolipoprotein A1
*APOC3*	406187	1.12	4.14*E* − 08	Apolipoprotein C3
*LCAT*	100303723	1.04	1.41*E* − 07	Lecithin-cholesterol acyltransferase
*PPARA*	397239	-1.67	1.66*E* − 13	Peroxisome proliferator-activated receptor alpha
*PPARGC1A*	397013	-0.99	0.001012566	PPARG coactivator 1 alpha
*CYP2E1*	403216	0.66	0.00042474	Cytochrome P450 family 2 subfamily E member 1
*GPX1*	397403	0.89	1.49*E* − 10	Glutathione peroxidase 1
*ROMO1*	100154394	1.16	2.48*E* − 08	Reactive oxygen species modulator 1
*TXNIP*	733688	-1.18	1.89*E* − 05	Thioredoxin interacting protein

Log2FoldChange of readcount by group H (H readcount) vs. group L (L readcount), of which 7 genes upregulated expression (log2FoldChange > 0) and 3 genes downregulated expression (log2FoldChange < 0).

## Data Availability

All data obtained in this study had been deposited to the National Center for Biotechnology Information (NCBI) with the accession number PRJNA721126 (https://www.ncbi.nlm.nih.gov/bioproject/PRJNA721126).

## Abbreviations

ABCA1: ATP-binding cassette subfamily A member 1
APOA1: Apolipoprotein A1
APOC3: Apolipoprotein C3
APOE: Apolipoprotein E
CM: Chylomicrons
CYP1A1: Cytochrome P450 family 1 subfamily A member 1
CYP2A6: Cytochrome P450 family 2 subfamily A member 6
CYP7A1: Cytochrome P450 family 7 subfamily A member 1
CYP26A1: Cytochrome P450 family 26 subfamily A member 1
CYP2E1: Cytochrome P450 family 2 subfamily E member 1
DEGs: Differentially expressed genes
FABP1: Fatty acid-binding protein 1
FAO: Fatty acids oxidation
FFA: Free fatty acids
FPKM: Fragments per kilobase of transcript sequence per million base-pairs sequenced
GAPDH: Glyceraldehyde-3-phosphate dehydrogenase
GO: Gene Ontology
GPX1: Glutathione peroxidase 1
HDL: High-density lipoproteins
KEGG: The Kyoto Encyclopedia of Genes and Genomes
LCAT: Lecithin-cholesterol acyltransferase
LCN2: Lipocalin-2
LDL: Low-density lipoproteins
LPL: Lipoprotein lipase
ndHDL: Nascent discoidal HDL
PCK1: Phosphoenolpyruvate carboxykinase 1
PPARA: Peroxisome proliferator-activated receptor alpha
PPARGC1A: PPARG coactivator 1 alpha
PPARs: Peroxisome proliferator-activated receptors
RCT: Reverse cholesterol transport
RetSat: Retinol saturase
ROMO1: ROS modulator 1
ROS: Reactive oxygen species
RNA-seq: RNA sequencing
SCOA: Succinyl-CoA : 3-ketoacid CoA-transferase
SEM: Standard error of mean
SRBI: Scavenger receptor class B typeI
TG: Triglycerides
TRX: Thioredoxin
TXNIP: Thioredoxin-interacting protein
VLDL: Very low-density lipoproteins.

## Supplementary Materials

Supplementary 1 Table S1: all enrichment terms in GO analysis.

Supplementary 2 Table S2: all enrichment pathways in KEGG analysis.
