# Supplementary material for: Differentially Expressed Hepatic Genes Revealed by Transcriptomics in Pigs with Different Liver Lipid Contents
Source: Oxid Med Cell Longev. 2022 Jan 28;2022:2315575. doi: 10.1155/2022/2315575 (PMC8817107; doi:10.1155/2022/2315575)
Supplement: Supplementary 1 — Table S1: all enrichment terms in GO analysis. [file 2315575.f1.docx]

| Category | GOID | Description | P-value | Gene Number | Gene Name |
| --- | --- | --- | --- | --- | --- |
| Biological process | GO:0044260 | cellular macromolecule metabolic process | 0.029411729 | 191 | TFRC/RPLP2/RPLP1/MIF/LOC100513261/SNRPA/RPS15/NME2/RPS20/AMbiological_process/RPL7A/SERPINA1/BGN/POLR2I/MSRB1/KIT/RPSA/UBA52/FAU/PSMB7/RACK1/GNAQ/APOA1/WNK1/OGT/UBE2D3/CMKLR1/NFKBIL1/GGTA1P/RPL29/RAMP1/HSPB1/RPL27/PSMB4/ERO1A/TGFBR2/COPS6/SRSF1/GAPDH/RPS21/SNRPD2/DCPS/CLU/KLF9/PRKAA1/RPS28/PRKAR2A/MEF2A/RPS9/PKIG/DDOST/RPS16/CSNK2B/RPS3/STAT1/MRPS18B/RPL10/CAST/TXNIP/NELFCD/RPL27A/ARAF/EEF1G/TIMP1/RPL13A/NR3C1/RABGGTA/RPS29/RTFDC1/RPL21/CDC42/IREB2/RPN1/PSMC5/SREBF1/HCST/TBRG4/IGF1R/AR/HMGB2/MGAT2/RPS23/RPS19/SOCS2/PPIA/UIMC1/RPL3/RSC1A1/ITGB1/RPL6/DPEP1/CSNK1A1/U2AF1/UCHL1/JAK2/TGFBR3/RNF114/RPL35/CAMK2D/CSTB/CD3D/PPARGC1A/ITIH1/RPS12/RPL31/Cellular componentL5/PSME1/PRKACA/BAG6/CDKN3/NGF/RNF19A/CTSB/IRF3/ADAM17/RPL15/OXSR1/CTSH/SOX9/IGF2/PSME2/PRNP/ADRA2A/THPO/DNASE1L1/Fbiological_process1/ZFX/C3/APP/RAP2B/CAPN3/MET/EIF2S3/VEGFA/UGP2/RENbiological_process/VCP/TRAPPC2/RTCB/RPL4/RPL22/GNRHR/GALNT1/FCER1G/ATM/IMPAD1/SUMO1/PIK3C3/DAD1/ENSA/TOP2A/ADM/LOC396905/MAN2B2/USP37/ITM2B/PRKAA2/VAPB/SPP1/DCN/NR5A1/PRKAR1A/ITIH4/GHR/NPC1/TFAM/HMGB1/MYLK/TSHZ2/RPS13/DDX39B/FGF1/IGF1/CD59/TLR9/FASLG/MOCS3/ABCF1/B3GNT5/ESR1/CDK4/ADCYAP1/TRAF6/PIK3CG/Cellular componentL25/FUT8/WT1/PLAGL1/MDH2/RBM4B/SERPINA6 |
| Biological process | GO:0044710 | single-organism metabolic process | 0.002150927 | 184 | TFRC/NAXE/GPX1/AK1/FAM213B/APOE/MIF/COX7A1/NME2/GPX4/IDH3G/Cellular componentS/APOC3/MSRB1/KIT/RACK1/COX5B/LCAT/APOA1/ADRB1/OGT/FAAH/GUK1/PRDX2/ETFB/HPD/ND3/GGTA1P/RAMP1/ERO1A/TGFBR2/GAPDH/PRKAA1/TALDO1/MEF2A/DDOST/NPL/PTGDS/RPS3/POR/AKR1A1/ATP5D/CYP2D25/ATP6V1A/ATP5G1/ARAF/ACY1/FUNDC2/TMEM86B/TPO/CDC42/IDH3A/GP91-PHOX/IYD/RPN1/PIP4K2A/GNAS/NAMPT/PSMC5/SREBF1/COX3/SUCLG2/TBRG4/IGF1R/MGAT2/COX7C/FTCD/ATP1B1/HSD17B8/IDH2/PDHA1/GULO/ITGB1/CYP2E1/DPEP1/ACMSD/UCHL1/EPX/DHRS4/UOX/LDHA/ENO3/CBR2/IMPA1/COX6C/PMVK/PPARGC1A/AHCY/Cellular componentL5/BAG6/DPYD/ATP5J/QDPR/ATP5I/ADAM17/GPT/COASY/CYP2C42/ADSS/OXSR1/OXCT1/CTSH/DIO2/SOX9/IGF2/CYBA/ADRA2A/PDXK/ACO2/FTH1/THPO/HADHA/GATM/Fbiological_process1/C3/APP/RAP2B/AFP/CYP3A29/VEGFA/UGP2/RENbiological_process/VCP/ATP8/UCP3/GALNT1/APOA4/FCER1G/ATM/FMO1/IMPAD1/PIK3C3/DAD1/GSTO1/ADM/MAN2B2/SDHA/GPI/RPIA/GOT1/SORD/PRKAA2/SPP1/ATP6/DHDH/NPC1/GPAM/CA3/CPT1B/ARG1/ETFDH/ABAT/TPI1/CRYL1/SUCLG1/AQP1/FGF1/TDH/HMGCR/IGF1/TLR9/MAOA/SCARB1/B3GNT5/ACADS/ESR1/CDK4/ACADL/ADCYAP1/GUCA2B/TRAF6/TXNRD1/PFKM/TKFC/PIK3CG/Cellular componentL25/FUT8/ENTPD1/BCAT2/VNN1/NEU1/MDH2/PSAP/MB21D1 |
| Biological process | GO:0006807 | nitrogen compound metabolic process | 0.021080534 | 180 | TFRC/PGLYRP2/NAXE/RPLP2/RPLP1/AK1/SNRPA/RPS15/NME2/RPS20/RPL7A/POLR2I/RPSA/UBA52/FAU/RACK1/COX5B/LCAT/APOA1/ADRB1/OGT/GUK1/CMKLR1/HPD/NFKBIL1/RPL29/RAMP1/RPL27/SRSF1/GAPDH/RPS21/SNRPD2/DCPS/CLU/KLF9/PRKAA1/TALDO1/RPS28/MEF2A/RPS9/PKIG/RPS16/RPS3/ATP5D/STAT1/MRPS18B/RPL10/ATP6V1A/ATP5G1/TXNIP/NELFCD/RPL27A/EEF1G/ACY1/RPL13A/NR3C1/TPO/RPS29/RTFDC1/RPL21/CDC42/IREB2/IYD/GNAS/NAMPT/PSMC5/SREBF1/AR/HMGB2/RPS23/COX7C/FTCD/ATP1B1/RPS19/UIMC1/RPL3/RSC1A1/RPL6/DPEP1/U2AF1/ACMSD/TGFBR3/RPL35/UOX/ENO3/CD3D/PPARGC1A/ITIH1/AHCY/RPS12/RPL31/DPYD/ATP5J/QDPR/ATP5I/IRF3/GPT/COASY/ADSS/RPL15/CTSH/DIO2/SOX9/IGF2/PRNP/ADRA2A/PDXK/COMT/SAT1/GATM/DNASE1L1/Fbiological_process1/ZFX/APP/CAPN3/MET/EIF2S3/HYAL1/VEGFA/UGP2/VCP/TRAPPC2/ATP8/RTCB/RPL4/RPL22/APOA4/ATM/FMO1/IMPAD1/SUMO1/TOP2A/GSTO1/ADM/SDHA/USP37/GPI/RPIA/GOT1/PRKAA2/VAPB/SPP1/NR5A1/ATP6/ITIH4/NLN/ARG1/TFAM/HMGB1/ABAT/TPI1/TSHZ2/RPS13/AQP1/DDX39B/FGF1/TDH/HMGCR/IGF1/TLR9/MAOA/FASLG/MOCS3/SCARB1/ABCF1/ESR1/SAT2/ADCYAP1/GUCA2B/TRAF6/PFKM/FUT8/BCAT2/WT1/PLAGL1/VNN1/NEU1/PSAP/MB21D1/RBM4B |
| Biological process | GO:0019538 | protein metabolic process | 0.010344363 | 170 | RPLP2/RPLP1/APOE/MIF/LOC100513261/RPS15/NME2/RPS20/AMbiological_process/RPL7A/SERPINA1/BGN/APOC3/MSRB1/KIT/RPSA/UBA52/FAU/PSMB7/RACK1/GNAQ/APOA1/WNK1/OGT/UBE2D3/GGTA1P/RPL29/RAMP1/HSPB1/RPL27/PSMB4/ERO1A/TGFBR2/COPS6/GAPDH/RPS21/CLU/PRKAA1/RPS28/PRKAR2A/MEF2A/CAPN7/RPS9/PKIG/DDOST/RPS16/CSNK2B/RPS3/STAT1/PROC/F2/MRPS18B/CAPNS1/RPL10/CAST/RPL27A/F12/ARAF/EEF1G/ACY1/TIMP1/RPL13A/RABGGTA/RPS29/CFB/RPL21/CDC42/IREB2/ADAM10/RPN1/PSMC5/HCST/TBRG4/IGF1R/MGAT2/RPS23/RPS19/SOCS2/PPIA/PLAU/UIMC1/RPL3/RPL6/CASP1/DPEP1/CSNK1A1/UCHL1/JAK2/RNF114/RPL35/CAMK2D/CFD/CSTB/DPP4/ITIH1/RPS12/RPL31/Cellular componentL5/PSME1/PRKACA/BAG6/PLG/CDKN3/NGF/RNF19A/CTSB/ADAM17/RPL15/OXSR1/CTSH/SOX9/IGF2/PSME2/PRNP/ADRA2A/THPO/C3/APP/RAP2B/CAPN3/MET/CASP3/EIF2S3/VEGFA/RENbiological_process/VCP/RPL4/RPL22/GNRHR/GALNT1/APOA4/ATM/IMPAD1/SUMO1/PIK3C3/DAD1/ENSA/LOC396905/MAN2B2/C1S/USP37/ITM2B/PRKAA2/VAPB/DCN/PRKAR1A/ITIH4/GHR/NPC1/NLN/MYLK/RPS13/FGF1/DESI2/CD59/TLR9/MOCS3/ABCF1/B3GNT5/ESR1/CDK4/ADCYAP1/TRAF6/PIK3CG/Cellular componentL25/FUT8/WT1/MDH2/RBM4B/SERPINA6 |
| Biological process | GO:0044267 | cellular protein metabolic process | 0.000852151 | 148 | RPLP2/RPLP1/MIF/LOC100513261/RPS15/NME2/RPS20/AMbiological_process/RPL7A/SERPINA1/BGN/MSRB1/KIT/RPSA/UBA52/FAU/PSMB7/RACK1/GNAQ/APOA1/WNK1/OGT/UBE2D3/GGTA1P/RPL29/RAMP1/HSPB1/RPL27/PSMB4/ERO1A/TGFBR2/COPS6/GAPDH/RPS21/CLU/PRKAA1/RPS28/PRKAR2A/MEF2A/RPS9/PKIG/DDOST/RPS16/CSNK2B/RPS3/STAT1/MRPS18B/RPL10/CAST/RPL27A/ARAF/EEF1G/TIMP1/RPL13A/RABGGTA/RPS29/RPL21/CDC42/IREB2/RPN1/PSMC5/HCST/TBRG4/IGF1R/MGAT2/RPS23/RPS19/SOCS2/PPIA/UIMC1/RPL3/RPL6/DPEP1/CSNK1A1/UCHL1/JAK2/RNF114/RPL35/CAMK2D/CSTB/ITIH1/RPS12/RPL31/Cellular componentL5/PSME1/PRKACA/BAG6/CDKN3/NGF/RNF19A/CTSB/ADAM17/RPL15/OXSR1/CTSH/SOX9/IGF2/PSME2/PRNP/ADRA2A/THPO/C3/APP/RAP2B/CAPN3/MET/EIF2S3/VEGFA/RENbiological_process/VCP/RPL4/RPL22/GNRHR/GALNT1/ATM/SUMO1/PIK3C3/DAD1/ENSA/LOC396905/MAN2B2/USP37/PRKAA2/VAPB/DCN/PRKAR1A/ITIH4/GHR/NPC1/MYLK/RPS13/FGF1/CD59/TLR9/MOCS3/ABCF1/B3GNT5/ESR1/CDK4/ADCYAP1/TRAF6/PIK3CG/Cellular componentL25/FUT8/WT1/MDH2/RBM4B/SERPINA6 |
| Biological process | GO:1901564 | organonitrogen compound metabolic process | 1.22165E-06 | 121 | PGLYRP2/NAXE/RPLP2/RPLP1/AK1/RPS15/NME2/RPS20/RPL7A/RPSA/UBA52/FAU/RACK1/COX5B/LCAT/APOA1/ADRB1/OGT/GUK1/HPD/RPL29/RAMP1/RPL27/GAPDH/RPS21/PRKAA1/TALDO1/RPS28/RPS9/RPS16/RPS3/ATP5D/MRPS18B/RPL10/ATP6V1A/ATP5G1/RPL27A/EEF1G/ACY1/RPL13A/TPO/RPS29/RPL21/IREB2/IYD/GNAS/NAMPT/RPS23/COX7C/FTCD/ATP1B1/RPS19/RPL3/RPL6/DPEP1/ACMSD/RPL35/UOX/ENO3/PPARGC1A/ITIH1/AHCY/RPS12/RPL31/DPYD/ATP5J/QDPR/ATP5I/GPT/COASY/ADSS/RPL15/CTSH/DIO2/PRNP/ADRA2A/PDXK/COMT/SAT1/GATM/Fbiological_process1/APP/EIF2S3/HYAL1/ATP8/RPL4/RPL22/APOA4/ATM/FMO1/IMPAD1/GSTO1/ADM/SDHA/GPI/RPIA/GOT1/ATP6/ITIH4/NLN/ARG1/ABAT/TPI1/RPS13/AQP1/TDH/HMGCR/MAOA/MOCS3/SCARB1/ABCF1/ESR1/SAT2/GUCA2B/PFKM/BCAT2/WT1/VNN1/NEU1/PSAP/RBM4B |
| Biological process | GO:0006793 | phosphorus metabolic process | 0.005514274 | 115 | NAXE/RPLP1/AK1/MIF/LOC100513261/NME2/BGN/KIT/RACK1/GNAQ/COX5B/LCAT/APOA1/WNK1/ADRB1/OGT/GUK1/RAMP1/HSPB1/TGFBR2/GAPDH/PRKAA1/TALDO1/PRKAR2A/MEF2A/PKIG/PPP1R14A/CSNK2B/RPS3/ATP5D/ATP6V1A/ATP5G1/ARAF/PPP1R14B/CDC42/PIP4K2A/GNAS/NAMPT/HCST/SUCLG2/TBRG4/IGF1R/AR/COX7C/ATP1B1/SOCS2/PDHA1/DPEP1/CSNK1A1/UCHL1/JAK2/ENO3/CAMK2D/IMPA1/PMVK/PPARGC1A/Cellular componentL5/PRKACA/PLPP1/CDKN3/ATP5J/ATP5I/ADAM17/COASY/ADSS/OXSR1/CTSH/SOX9/PRNP/ADRA2A/PDXK/THPO/Fbiological_process1/C3/APP/RAP2B/MET/VEGFA/UGP2/ATP8/APOA4/ATM/FMO1/IMPAD1/PIK3C3/ENSA/ADM/SDHA/GPI/RPIA/PRKAA2/VAPB/DCN/PRKAR1A/ATP6/GHR/GPAM/MYLK/TPI1/AQP1/FGF1/HMGCR/TLR9/MOCS3/SCARB1/CDK4/ADCYAP1/GUCA2B/TRAF6/PFKM/TKFC/PIK3CG/Cellular componentL25/FUT8/MB21D1 |
| Biological process | GO:0006796 | phosphate-containing compound metabolic process | 0.011514046 | 111 | NAXE/RPLP1/AK1/MIF/LOC100513261/NME2/BGN/KIT/RACK1/GNAQ/COX5B/LCAT/APOA1/WNK1/ADRB1/OGT/GUK1/RAMP1/HSPB1/TGFBR2/GAPDH/PRKAA1/TALDO1/PRKAR2A/MEF2A/PKIG/PPP1R14A/CSNK2B/RPS3/ATP5D/ATP6V1A/ATP5G1/ARAF/PPP1R14B/CDC42/PIP4K2A/GNAS/NAMPT/HCST/TBRG4/IGF1R/AR/COX7C/ATP1B1/SOCS2/DPEP1/CSNK1A1/UCHL1/JAK2/ENO3/CAMK2D/IMPA1/PMVK/PPARGC1A/Cellular componentL5/PRKACA/PLPP1/CDKN3/ATP5J/ATP5I/ADAM17/COASY/ADSS/OXSR1/CTSH/SOX9/PRNP/ADRA2A/PDXK/THPO/Fbiological_process1/C3/APP/RAP2B/MET/VEGFA/ATP8/APOA4/ATM/FMO1/IMPAD1/PIK3C3/ENSA/ADM/SDHA/GPI/RPIA/PRKAA2/VAPB/DCN/PRKAR1A/ATP6/GHR/GPAM/MYLK/TPI1/AQP1/FGF1/HMGCR/TLR9/MOCS3/SCARB1/CDK4/ADCYAP1/GUCA2B/TRAF6/PFKM/TKFC/PIK3CG/Cellular componentL25/MB21D1 |
| Biological process | GO:0044281 | small molecule metabolic process | 0.002347212 | 109 | NAXE/AK1/FAM213B/APOE/MIF/NME2/IDH3G/APOC3/RACK1/COX5B/LCAT/APOA1/ADRB1/OGT/FAAH/GUK1/ETFB/HPD/RAMP1/GAPDH/PRKAA1/TALDO1/NPL/PTGDS/ATP5D/CYP2D25/ATP6V1A/ATP5G1/ACY1/TMEM86B/IDH3A/IYD/GNAS/NAMPT/SREBF1/SUCLG2/COX7C/FTCD/ATP1B1/HSD17B8/IDH2/PDHA1/GULO/CYP2E1/DPEP1/ACMSD/UOX/LDHA/ENO3/IMPA1/PMVK/PPARGC1A/AHCY/DPYD/ATP5J/ATP5I/GPT/COASY/CYP2C42/ADSS/OXCT1/IGF2/ADRA2A/PDXK/ACO2/HADHA/GATM/Fbiological_process1/C3/APP/AFP/RENbiological_process/ATP8/APOA4/FMO1/IMPAD1/GSTO1/ADM/MAN2B2/SDHA/GPI/RPIA/GOT1/PRKAA2/ATP6/DHDH/NPC1/CA3/CPT1B/ARG1/ABAT/TPI1/CRYL1/SUCLG1/AQP1/FGF1/TDH/HMGCR/IGF1/SCARB1/ACADS/ACADL/GUCA2B/PFKM/TKFC/BCAT2/VNN1/MDH2/MB21D1 |
| Biological process | GO:0043412 | macromolecule modification | 0.029165918 | 92 | RPLP1/MIF/LOC100513261/NME2/AMbiological_process/BGN/KIT/RACK1/GNAQ/APOA1/WNK1/OGT/UBE2D3/GGTA1P/RAMP1/HSPB1/ERO1A/TGFBR2/COPS6/GAPDH/CLU/PRKAA1/PRKAR2A/MEF2A/PKIG/DDOST/CSNK2B/RPS3/ARAF/NR3C1/RABGGTA/CDC42/RPN1/HCST/TBRG4/IGF1R/MGAT2/SOCS2/PPIA/UIMC1/CSNK1A1/UCHL1/JAK2/RNF114/CAMK2D/Cellular componentL5/PRKACA/BAG6/CDKN3/RNF19A/ADAM17/OXSR1/CTSH/SOX9/IGF2/PRNP/ADRA2A/THPO/C3/APP/RAP2B/CAPN3/MET/VEGFA/VCP/GNRHR/GALNT1/ATM/SUMO1/PIK3C3/DAD1/ENSA/MAN2B2/USP37/PRKAA2/VAPB/DCN/PRKAR1A/GHR/NPC1/MYLK/FGF1/TLR9/MOCS3/B3GNT5/CDK4/ADCYAP1/TRAF6/PIK3CG/Cellular componentL25/FUT8/MDH2 |
| Biological process | GO:0006464 | cellular protein modification process | 0.026078517 | 91 | RPLP1/MIF/LOC100513261/NME2/AMbiological_process/BGN/KIT/RACK1/GNAQ/APOA1/WNK1/OGT/UBE2D3/GGTA1P/RAMP1/HSPB1/ERO1A/TGFBR2/COPS6/GAPDH/CLU/PRKAA1/PRKAR2A/MEF2A/PKIG/DDOST/CSNK2B/RPS3/ARAF/RABGGTA/CDC42/RPN1/HCST/TBRG4/IGF1R/MGAT2/SOCS2/PPIA/UIMC1/CSNK1A1/UCHL1/JAK2/RNF114/CAMK2D/Cellular componentL5/PRKACA/BAG6/CDKN3/RNF19A/ADAM17/OXSR1/CTSH/SOX9/IGF2/PRNP/ADRA2A/THPO/C3/APP/RAP2B/CAPN3/MET/VEGFA/VCP/GNRHR/GALNT1/ATM/SUMO1/PIK3C3/DAD1/ENSA/MAN2B2/USP37/PRKAA2/VAPB/DCN/PRKAR1A/GHR/NPC1/MYLK/FGF1/TLR9/MOCS3/B3GNT5/CDK4/ADCYAP1/TRAF6/PIK3CG/Cellular componentL25/FUT8/MDH2 |
| Biological process | GO:0036211 | protein modification process | 0.026078517 | 91 | RPLP1/MIF/LOC100513261/NME2/AMbiological_process/BGN/KIT/RACK1/GNAQ/APOA1/WNK1/OGT/UBE2D3/GGTA1P/RAMP1/HSPB1/ERO1A/TGFBR2/COPS6/GAPDH/CLU/PRKAA1/PRKAR2A/MEF2A/PKIG/DDOST/CSNK2B/RPS3/ARAF/RABGGTA/CDC42/RPN1/HCST/TBRG4/IGF1R/MGAT2/SOCS2/PPIA/UIMC1/CSNK1A1/UCHL1/JAK2/RNF114/CAMK2D/Cellular componentL5/PRKACA/BAG6/CDKN3/RNF19A/ADAM17/OXSR1/CTSH/SOX9/IGF2/PRNP/ADRA2A/THPO/C3/APP/RAP2B/CAPN3/MET/VEGFA/VCP/GNRHR/GALNT1/ATM/SUMO1/PIK3C3/DAD1/ENSA/MAN2B2/USP37/PRKAA2/VAPB/DCN/PRKAR1A/GHR/NPC1/MYLK/FGF1/TLR9/MOCS3/B3GNT5/CDK4/ADCYAP1/TRAF6/PIK3CG/Cellular componentL25/FUT8/MDH2 |
| Biological process | GO:0016310 | phosphorylation | 0.030927314 | 77 | RPLP1/AK1/MIF/NME2/BGN/KIT/RACK1/GNAQ/COX5B/WNK1/OGT/GUK1/HSPB1/TGFBR2/GAPDH/PRKAA1/PRKAR2A/MEF2A/PKIG/PPP1R14A/CSNK2B/RPS3/ARAF/PPP1R14B/CDC42/PIP4K2A/HCST/TBRG4/IGF1R/AR/COX7C/SOCS2/CSNK1A1/UCHL1/JAK2/ENO3/CAMK2D/IMPA1/PMVK/Cellular componentL5/PRKACA/ADAM17/COASY/OXSR1/CTSH/SOX9/PRNP/ADRA2A/PDXK/THPO/Fbiological_process1/C3/APP/RAP2B/MET/VEGFA/ATM/IMPAD1/PIK3C3/SDHA/GPI/PRKAA2/VAPB/DCN/PRKAR1A/GHR/MYLK/TPI1/FGF1/TLR9/CDK4/ADCYAP1/TRAF6/PFKM/TKFC/PIK3CG/Cellular componentL25 |
| Biological process | GO:0009056 | catabolic process | 0.01899215 | 76 | PGLYRP2/APOE/APOC3/PSMB7/RACK1/APOA1/OGT/FAAH/ETFB/HPD/PSMB4/GAPDH/DCPS/CLU/PRKAA1/NPL/CAST/ARAF/FUNDC2/TIMP1/TPO/CDC42/PSMC5/FTCD/ITGB1/UCHL1/EPX/RNF114/UOX/ENO3/IMPA1/AHCY/PSME1/BAG6/RNF19A/DPYD/LEPR/CTSB/GPT/OXCT1/CTSH/PSME2/ADRA2A/COMT/SAT1/HADHA/DNASE1L1/Fbiological_process1/HYAL1/RENbiological_process/VCP/GNRHR/APOA4/ATM/SUMO1/PIK3C3/GSTO1/USP37/GPI/GOT1/PRKAA2/SPP1/DHDH/CPT1B/HMGB1/ABAT/TPI1/BECN1/TDH/MAOA/SCARB1/CDK4/ACADL/SAT2/PFKM/NEU1 |
| Biological process | GO:1901566 | organonitrogen compound biosynthetic process | 0.000451815 | 72 | RPLP2/RPLP1/RPS15/NME2/RPS20/RPL7A/RPSA/UBA52/FAU/RACK1/COX5B/ADRB1/RPL29/RAMP1/RPL27/GAPDH/RPS21/RPS28/RPS9/RPS16/RPS3/ATP5D/MRPS18B/RPL10/ATP5G1/RPL27A/EEF1G/RPL13A/RPS29/RPL21/IREB2/GNAS/NAMPT/RPS23/RPS19/RPL3/RPL6/ACMSD/RPL35/PPARGC1A/RPS12/RPL31/DPYD/ATP5J/QDPR/ATP5I/COASY/ADSS/RPL15/ADRA2A/PDXK/GATM/APP/EIF2S3/HYAL1/ATP8/RPL4/RPL22/ATM/ADM/GOT1/ATP6/ARG1/RPS13/AQP1/MOCS3/ABCF1/ESR1/GUCA2B/BCAT2/WT1/RBM4B |
| Biological process | GO:0044085 | cellular component biogenesis | 0.011170806 | 66 | APOE/MIF/RPS15/RPL7A/APOC3/MSRB1/KIT/RPSA/RACK1/APOA1/TLR4/LIMA1/PET100/RPL27/SRSF1/RPS21/SNRPD2/CLU/RPS28/MEF2A/RPS16/RPS3/PPP2R1A/RPL10/RDX/RPLP0/CAPZA2/RPL13A/CDC42/COX17/PSMC5/MX2/COX3/HMGB2/RPS19/HSD17B8/RPL3/RPL6/RPL35/CD3D/RAB34/BAG6/SOX9/PRNP/Fbiological_process1/APP/CAPN3/MET/HYAL1/VEGFA/APOA4/FCER1G/SUMO1/PIK3C3/TFAM/HMGB1/DDX39B/BECN1/AIF1/RAB11A/ABCF1/SLC9A1/ESR1/NTN1/VCL/NECTIN1 |
| Biological process | GO:1901575 | organic substance catabolic process | 0.028055262 | 64 | PGLYRP2/APOE/APOC3/PSMB7/RACK1/APOA1/OGT/FAAH/ETFB/HPD/PSMB4/GAPDH/DCPS/CLU/PRKAA1/NPL/CAST/ARAF/TIMP1/CDC42/PSMC5/FTCD/UCHL1/RNF114/UOX/ENO3/IMPA1/AHCY/PSME1/BAG6/RNF19A/DPYD/CTSB/GPT/OXCT1/CTSH/PSME2/ADRA2A/SAT1/HADHA/DNASE1L1/Fbiological_process1/HYAL1/RENbiological_process/VCP/GNRHR/APOA4/ATM/SUMO1/USP37/GPI/GOT1/SPP1/DHDH/CPT1B/ABAT/TPI1/TDH/SCARB1/CDK4/ACADL/SAT2/PFKM/NEU1 |
| Biological process | GO:0006082 | organic acid metabolic process | 0.003989175 | 62 | FAM213B/MIF/IDH3G/APOC3/APOA1/OGT/FAAH/ETFB/HPD/GAPDH/PRKAA1/NPL/PTGDS/CYP2D25/ACY1/IDH3A/IYD/SUCLG2/FTCD/HSD17B8/IDH2/PDHA1/GULO/CYP2E1/DPEP1/ACMSD/UOX/LDHA/ENO3/PPARGC1A/AHCY/DPYD/GPT/CYP2C42/ACO2/HADHA/GATM/Fbiological_process1/C3/RENbiological_process/APOA4/FMO1/IMPAD1/GSTO1/SDHA/GPI/GOT1/PRKAA2/NPC1/CPT1B/ARG1/ABAT/TPI1/CRYL1/SUCLG1/TDH/ACADS/ACADL/PFKM/BCAT2/VNN1/MDH2 |
| Biological process | GO:1901135 | carbohydrate derivative metabolic process | 0.005684583 | 60 | PGLYRP2/AK1/NME2/RACK1/COX5B/ADRB1/OGT/GUK1/GGTA1P/RAMP1/GAPDH/PRKAA1/TALDO1/DDOST/NPL/ATP5D/ATP6V1A/ATP5G1/RPN1/GNAS/MGAT2/COX7C/ATP1B1/DPEP1/ENO3/PPARGC1A/ITIH1/AHCY/DPYD/ATP5J/ATP5I/COASY/ADSS/ADRA2A/Fbiological_process1/HYAL1/UGP2/RENbiological_process/VCP/ATP8/GALNT1/IMPAD1/DAD1/ADM/MAN2B2/SDHA/GPI/RPIA/ITM2B/ATP6/ITIH4/NPC1/TPI1/AQP1/HMGCR/B3GNT5/GUCA2B/PFKM/FUT8/NEU1 |
| Biological process | GO:0043436 | oxoacid metabolic process | 0.007333956 | 60 | FAM213B/MIF/IDH3G/APOC3/APOA1/OGT/FAAH/ETFB/HPD/GAPDH/PRKAA1/NPL/PTGDS/CYP2D25/ACY1/IDH3A/IYD/SUCLG2/FTCD/HSD17B8/IDH2/PDHA1/GULO/CYP2E1/DPEP1/ACMSD/LDHA/ENO3/PPARGC1A/AHCY/DPYD/GPT/CYP2C42/ACO2/HADHA/GATM/Fbiological_process1/C3/RENbiological_process/APOA4/IMPAD1/GSTO1/SDHA/GPI/GOT1/PRKAA2/NPC1/CPT1B/ARG1/ABAT/TPI1/CRYL1/SUCLG1/TDH/ACADS/ACADL/PFKM/BCAT2/VNN1/MDH2 |
| Biological process | GO:0019752 | carboxylic acid metabolic process | 0.00982213 | 59 | FAM213B/MIF/IDH3G/APOC3/APOA1/OGT/FAAH/ETFB/HPD/GAPDH/PRKAA1/NPL/PTGDS/CYP2D25/ACY1/IDH3A/IYD/SUCLG2/FTCD/HSD17B8/IDH2/PDHA1/GULO/CYP2E1/DPEP1/ACMSD/LDHA/ENO3/PPARGC1A/AHCY/DPYD/GPT/CYP2C42/ACO2/HADHA/GATM/Fbiological_process1/C3/RENbiological_process/APOA4/GSTO1/SDHA/GPI/GOT1/PRKAA2/NPC1/CPT1B/ARG1/ABAT/TPI1/CRYL1/SUCLG1/TDH/ACADS/ACADL/PFKM/BCAT2/VNN1/MDH2 |
| Biological process | GO:0022607 | cellular component assembly | 0.00982213 | 59 | APOE/MIF/RPS15/APOC3/MSRB1/KIT/RPSA/RACK1/APOA1/TLR4/LIMA1/PET100/SRSF1/SNRPD2/CLU/RPS28/MEF2A/RPS3/PPP2R1A/RPL10/RDX/CAPZA2/RPL13A/CDC42/COX17/PSMC5/MX2/COX3/HMGB2/RPS19/HSD17B8/RPL3/RPL6/CD3D/RAB34/BAG6/SOX9/PRNP/Fbiological_process1/APP/CAPN3/MET/HYAL1/VEGFA/APOA4/FCER1G/SUMO1/PIK3C3/TFAM/HMGB1/DDX39B/BECN1/AIF1/RAB11A/SLC9A1/ESR1/NTN1/VCL/NECTIN1 |
| Biological process | GO:0044248 | cellular catabolic process | 0.042880045 | 59 | APOE/APOC3/PSMB7/RACK1/APOA1/FAAH/ETFB/HPD/PSMB4/DCPS/CLU/PRKAA1/NPL/ARAF/FUNDC2/TIMP1/TPO/PSMC5/FTCD/UCHL1/EPX/RNF114/UOX/AHCY/PSME1/BAG6/RNF19A/DPYD/LEPR/CTSB/GPT/OXCT1/CTSH/PSME2/ADRA2A/COMT/HADHA/DNASE1L1/RENbiological_process/VCP/GNRHR/APOA4/ATM/SUMO1/PIK3C3/GSTO1/USP37/GOT1/PRKAA2/CPT1B/HMGB1/ABAT/TPI1/BECN1/TDH/MAOA/SCARB1/ACADL/NEU1 |
| Biological process | GO:0019637 | organophosphate metabolic process | 0.011293729 | 56 | NAXE/AK1/NME2/RACK1/COX5B/LCAT/APOA1/ADRB1/OGT/GUK1/RAMP1/GAPDH/PRKAA1/TALDO1/ATP5D/ATP6V1A/ATP5G1/CDC42/PIP4K2A/GNAS/NAMPT/COX7C/ATP1B1/DPEP1/ENO3/IMPA1/PMVK/PPARGC1A/ATP5J/ATP5I/COASY/ADSS/ADRA2A/PDXK/Fbiological_process1/ATP8/APOA4/ATM/FMO1/IMPAD1/PIK3C3/ADM/SDHA/GPI/RPIA/ATP6/GPAM/TPI1/AQP1/HMGCR/MOCS3/SCARB1/GUCA2B/PFKM/PIK3CG/MB21D1 |
| Biological process | GO:0043603 | cellular amide metabolic process | 0.00094699 | 51 | RPLP2/RPLP1/RPS15/RPS20/RPL7A/RPSA/UBA52/FAU/RACK1/RPL29/RPL27/GAPDH/RPS21/RPS28/RPS9/RPS16/RPS3/MRPS18B/RPL10/RPL27A/EEF1G/RPL13A/RPS29/RPL21/IREB2/RPS23/FTCD/RPS19/RPL3/RPL6/RPL35/RPS12/RPL31/RPL15/CTSH/PRNP/APP/EIF2S3/RPL4/RPL22/ATM/GSTO1/NLN/ARG1/RPS13/ABCF1/ESR1/WT1/VNN1/NEU1/RBM4B |
| Biological process | GO:0043933 | macromolecular complex subunit organization | 0.009252593 | 49 | APOE/MIF/RPS15/APOC3/MSRB1/RPSA/RACK1/APOA1/TLR4/LIMA1/PET100/SRSF1/SNRPD2/CLU/RPS28/RPS3/PPP2R1A/RPL10/RDX/CAPZA2/RPL13A/CDC42/COX17/PSMC5/MX2/COX3/CFL1/HMGB2/RPS19/HSD17B8/RPL3/RPL6/CD3D/SOX9/PRNP/Fbiological_process1/CAPN3/MET/VEGFA/APOA4/FCER1G/SUMO1/TFAM/HMGB1/DDX39B/AIF1/SCARB1/SLC9A1/ESR1 |
| Biological process | GO:0006518 | peptide metabolic process | 0.00071054 | 47 | RPLP2/RPLP1/RPS15/RPS20/RPL7A/RPSA/UBA52/FAU/RACK1/RPL29/RPL27/GAPDH/RPS21/RPS28/RPS9/RPS16/RPS3/MRPS18B/RPL10/RPL27A/EEF1G/RPL13A/RPS29/RPL21/IREB2/RPS23/RPS19/RPL3/RPL6/RPL35/RPS12/RPL31/RPL15/CTSH/PRNP/APP/EIF2S3/RPL4/RPL22/ATM/GSTO1/NLN/RPS13/ABCF1/ESR1/WT1/RBM4B |
| Biological process | GO:0065003 | macromolecular complex assembly | 0.00311487 | 46 | APOE/MIF/RPS15/APOC3/MSRB1/RPSA/RACK1/APOA1/TLR4/PET100/SRSF1/SNRPD2/CLU/RPS28/RPS3/PPP2R1A/RPL10/RDX/CAPZA2/RPL13A/CDC42/COX17/PSMC5/MX2/COX3/HMGB2/RPS19/HSD17B8/RPL3/RPL6/CD3D/SOX9/PRNP/Fbiological_process1/CAPN3/MET/VEGFA/APOA4/FCER1G/SUMO1/TFAM/HMGB1/DDX39B/AIF1/SLC9A1/ESR1 |
| Biological process | GO:0043604 | amide biosynthetic process | 0.000313753 | 44 | RPLP2/RPLP1/RPS15/RPS20/RPL7A/RPSA/UBA52/FAU/RACK1/RPL29/RPL27/GAPDH/RPS21/RPS28/RPS9/RPS16/RPS3/MRPS18B/RPL10/RPL27A/EEF1G/RPL13A/RPS29/RPL21/IREB2/RPS23/RPS19/RPL3/RPL6/RPL35/RPS12/RPL31/RPL15/APP/EIF2S3/RPL4/RPL22/ATM/ARG1/RPS13/ABCF1/ESR1/WT1/RBM4B |
| Biological process | GO:0006412 | translation | 4.11603E-05 | 43 | RPLP2/RPLP1/RPS15/RPS20/RPL7A/RPSA/UBA52/FAU/RACK1/RPL29/RPL27/GAPDH/RPS21/RPS28/RPS9/RPS16/RPS3/MRPS18B/RPL10/RPL27A/EEF1G/RPL13A/RPS29/RPL21/IREB2/RPS23/RPS19/RPL3/RPL6/RPL35/RPS12/RPL31/RPL15/APP/EIF2S3/RPL4/RPL22/ATM/RPS13/ABCF1/ESR1/WT1/RBM4B |
| Biological process | GO:0043043 | peptide biosynthetic process | 7.09755E-05 | 43 | RPLP2/RPLP1/RPS15/RPS20/RPL7A/RPSA/UBA52/FAU/RACK1/RPL29/RPL27/GAPDH/RPS21/RPS28/RPS9/RPS16/RPS3/MRPS18B/RPL10/RPL27A/EEF1G/RPL13A/RPS29/RPL21/IREB2/RPS23/RPS19/RPL3/RPL6/RPL35/RPS12/RPL31/RPL15/APP/EIF2S3/RPL4/RPL22/ATM/RPS13/ABCF1/ESR1/WT1/RBM4B |
| Biological process | GO:0044712 | single-organism catabolic process | 0.01191004 | 43 | APOE/APOC3/RACK1/APOA1/OGT/FAAH/ETFB/HPD/GAPDH/PRKAA1/NPL/FUNDC2/PSMC5/FTCD/ITGB1/UOX/ENO3/IMPA1/AHCY/BAG6/DPYD/GPT/OXCT1/ADRA2A/HADHA/Fbiological_process1/RENbiological_process/VCP/APOA4/GSTO1/GPI/GOT1/SPP1/DHDH/CPT1B/ABAT/TPI1/TDH/SCARB1/CDK4/ACADL/PFKM/NEU1 |
| Biological process | GO:0055086 | nucleobase-containing small molecule metabolic process | 0.020374708 | 43 | NAXE/AK1/NME2/RACK1/COX5B/ADRB1/OGT/GUK1/RAMP1/GAPDH/PRKAA1/TALDO1/ATP5D/ATP6V1A/ATP5G1/GNAS/NAMPT/COX7C/ATP1B1/UOX/ENO3/PPARGC1A/AHCY/DPYD/ATP5J/ATP5I/COASY/ADSS/ADRA2A/Fbiological_process1/ATP8/FMO1/ADM/SDHA/GPI/RPIA/ATP6/TPI1/AQP1/HMGCR/GUCA2B/PFKM/MB21D1 |
| Biological process | GO:0006753 | nucleoside phosphate metabolic process | 0.029944475 | 40 | NAXE/AK1/NME2/RACK1/COX5B/ADRB1/OGT/GUK1/RAMP1/GAPDH/PRKAA1/TALDO1/ATP5D/ATP6V1A/ATP5G1/GNAS/NAMPT/COX7C/ATP1B1/ENO3/PPARGC1A/ATP5J/ATP5I/COASY/ADSS/ADRA2A/Fbiological_process1/ATP8/FMO1/ADM/SDHA/GPI/RPIA/ATP6/TPI1/AQP1/HMGCR/GUCA2B/PFKM/MB21D1 |
| Biological process | GO:0009117 | nucleotide metabolic process | 0.029944475 | 40 | NAXE/AK1/NME2/RACK1/COX5B/ADRB1/OGT/GUK1/RAMP1/GAPDH/PRKAA1/TALDO1/ATP5D/ATP6V1A/ATP5G1/GNAS/NAMPT/COX7C/ATP1B1/ENO3/PPARGC1A/ATP5J/ATP5I/COASY/ADSS/ADRA2A/Fbiological_process1/ATP8/FMO1/ADM/SDHA/GPI/RPIA/ATP6/TPI1/AQP1/HMGCR/GUCA2B/PFKM/MB21D1 |
| Biological process | GO:0044255 | cellular lipid metabolic process | 0.029944475 | 40 | FAM213B/APOE/MIF/APOC3/LCAT/APOA1/FAAH/ETFB/GGTA1P/PRKAA1/PTGDS/CYP2D25/TMEM86B/CDC42/PIP4K2A/SREBF1/HSD17B8/CYP2E1/DPEP1/IMPA1/PMVK/PPARGC1A/CYP2C42/HADHA/C3/APOA4/ATM/IMPAD1/PIK3C3/PRKAA2/GPAM/CPT1B/CRYL1/HMGCR/SCARB1/ACADS/ACADL/PIK3CG/NEU1/PSAP |
| Biological process | GO:0048585 | negative regulation of response to stimulus | 0.034289424 | 37 | MIF/LOC100513261/BGN/RACK1/NFKBIL1/HSPB1/CLU/PRKAA1/RPS3/STAT1/PROC/F12/ARAF/LMNA/IGF1R/AR/HMGB2/LEPROT/SOCS2/UCHL1/TMEM14A/PLG/SOX9/PRNP/ADRA2A/Fbiological_process1/MET/ADM/PRKAA2/DCN/ARG1/BCL2L1/DDX39B/IGF1/CD59/ESR1/TKFC |
| Biological process | GO:0019693 | ribose phosphate metabolic process | 0.021047679 | 36 | AK1/NME2/RACK1/COX5B/ADRB1/OGT/GUK1/RAMP1/GAPDH/PRKAA1/TALDO1/ATP5D/ATP6V1A/ATP5G1/GNAS/COX7C/ATP1B1/ENO3/PPARGC1A/ATP5J/ATP5I/COASY/ADSS/ADRA2A/Fbiological_process1/ATP8/ADM/SDHA/GPI/RPIA/ATP6/TPI1/AQP1/HMGCR/GUCA2B/PFKM |
| Biological process | GO:0072521 | purine-containing compound metabolic process | 0.035867144 | 36 | AK1/NME2/RACK1/COX5B/ADRB1/OGT/GUK1/RAMP1/GAPDH/PRKAA1/ATP5D/ATP6V1A/ATP5G1/GNAS/COX7C/ATP1B1/UOX/ENO3/PPARGC1A/AHCY/ATP5J/ATP5I/COASY/ADSS/ADRA2A/Fbiological_process1/ATP8/ADM/SDHA/GPI/ATP6/TPI1/AQP1/HMGCR/GUCA2B/PFKM |
| Biological process | GO:0032787 | monocarboxylic acid metabolic process | 0.00284337 | 34 | FAM213B/MIF/APOC3/APOA1/OGT/FAAH/ETFB/GAPDH/PRKAA1/PTGDS/CYP2D25/FTCD/HSD17B8/IDH2/PDHA1/CYP2E1/ENO3/PPARGC1A/CYP2C42/HADHA/Fbiological_process1/C3/APOA4/GPI/PRKAA2/NPC1/CPT1B/ABAT/TPI1/CRYL1/ACADS/ACADL/PFKM/VNN1 |
| Biological process | GO:0009150 | purine ribonucleotide metabolic process | 0.030179246 | 34 | AK1/NME2/RACK1/COX5B/ADRB1/OGT/GUK1/RAMP1/GAPDH/PRKAA1/ATP5D/ATP6V1A/ATP5G1/GNAS/COX7C/ATP1B1/ENO3/PPARGC1A/ATP5J/ATP5I/COASY/ADSS/ADRA2A/Fbiological_process1/ATP8/ADM/SDHA/GPI/ATP6/TPI1/AQP1/HMGCR/GUCA2B/PFKM |
| Biological process | GO:0009259 | ribonucleotide metabolic process | 0.030179246 | 34 | AK1/NME2/RACK1/COX5B/ADRB1/OGT/GUK1/RAMP1/GAPDH/PRKAA1/ATP5D/ATP6V1A/ATP5G1/GNAS/COX7C/ATP1B1/ENO3/PPARGC1A/ATP5J/ATP5I/COASY/ADSS/ADRA2A/Fbiological_process1/ATP8/ADM/SDHA/GPI/ATP6/TPI1/AQP1/HMGCR/GUCA2B/PFKM |
| Biological process | GO:0006163 | purine nucleotide metabolic process | 0.039232984 | 34 | AK1/NME2/RACK1/COX5B/ADRB1/OGT/GUK1/RAMP1/GAPDH/PRKAA1/ATP5D/ATP6V1A/ATP5G1/GNAS/COX7C/ATP1B1/ENO3/PPARGC1A/ATP5J/ATP5I/COASY/ADSS/ADRA2A/Fbiological_process1/ATP8/ADM/SDHA/GPI/ATP6/TPI1/AQP1/HMGCR/GUCA2B/PFKM |
| Biological process | GO:0005975 | carbohydrate metabolic process | 0.018250845 | 32 | OGT/GGTA1P/GAPDH/PRKAA1/TALDO1/NPL/HEXB/MGAT2/IDH2/PDHA1/GULO/LDHA/ENO3/IMPA1/IGF2/Fbiological_process1/HYAL1/UGP2/IMPAD1/GSTO1/MAN2B2/GPI/RPIA/GOT1/DHDH/TPI1/IGF1/PFKM/TKFC/ENTPD1/NEU1/MDH2 |
| Biological process | GO:0051338 | regulation of transferase activity | 0.013618379 | 31 | RPLP1/MIF/LOC100513261/BGN/KIT/RACK1/GNAQ/WNK1/HSPB1/PRKAR2A/PKIG/CSNK2B/RPS3/ARAF/CDC42/IGF1R/SOCS2/UCHL1/JAK2/OXSR1/PRNP/ADRA2A/APP/RAP2B/VEGFA/VAPB/DCN/PRKAR1A/FGF1/ADCYAP1/TRAF6 |
| Biological process | GO:0009968 | negative regulation of signal transduction | 0.006707784 | 30 | MIF/LOC100513261/BGN/RACK1/NFKBIL1/HSPB1/CLU/PRKAA1/STAT1/ARAF/IGF1R/AR/HMGB2/LEPROT/SOCS2/UCHL1/TMEM14A/SOX9/PRNP/ADRA2A/Fbiological_process1/MET/ADM/PRKAA2/DCN/ARG1/BCL2L1/IGF1/ESR1/TKFC |
| Biological process | GO:0043549 | regulation of kinase activity | 0.014029011 | 30 | RPLP1/MIF/BGN/KIT/RACK1/GNAQ/WNK1/HSPB1/PRKAR2A/PKIG/CSNK2B/RPS3/ARAF/CDC42/IGF1R/SOCS2/UCHL1/JAK2/OXSR1/PRNP/ADRA2A/APP/RAP2B/VEGFA/VAPB/DCN/PRKAR1A/FGF1/ADCYAP1/TRAF6 |
| Biological process | GO:0045859 | regulation of protein kinase activity | 0.014029011 | 30 | RPLP1/MIF/BGN/KIT/RACK1/GNAQ/WNK1/HSPB1/PRKAR2A/PKIG/CSNK2B/RPS3/ARAF/CDC42/IGF1R/SOCS2/UCHL1/JAK2/OXSR1/PRNP/ADRA2A/APP/RAP2B/VEGFA/VAPB/DCN/PRKAR1A/FGF1/ADCYAP1/TRAF6 |
| Biological process | GO:0010648 | negative regulation of cell communication | 0.026724258 | 30 | MIF/LOC100513261/BGN/RACK1/NFKBIL1/HSPB1/CLU/PRKAA1/STAT1/ARAF/IGF1R/AR/HMGB2/LEPROT/SOCS2/UCHL1/TMEM14A/SOX9/PRNP/ADRA2A/Fbiological_process1/MET/ADM/PRKAA2/DCN/ARG1/BCL2L1/IGF1/ESR1/TKFC |
| Biological process | GO:0023057 | negative regulation of signaling | 0.026724258 | 30 | MIF/LOC100513261/BGN/RACK1/NFKBIL1/HSPB1/CLU/PRKAA1/STAT1/ARAF/IGF1R/AR/HMGB2/LEPROT/SOCS2/UCHL1/TMEM14A/SOX9/PRNP/ADRA2A/Fbiological_process1/MET/ADM/PRKAA2/DCN/ARG1/BCL2L1/IGF1/ESR1/TKFC |
| Biological process | GO:0006461 | protein complex assembly | 0.046887733 | 30 | MIF/MSRB1/RACK1/TLR4/PET100/CLU/RPS3/PPP2R1A/RDX/CAPZA2/CDC42/COX17/PSMC5/MX2/COX3/HSD17B8/CD3D/SOX9/PRNP/Fbiological_process1/CAPN3/MET/VEGFA/FCER1G/SUMO1/TFAM/HMGB1/AIF1/SLC9A1/ESR1 |
| Biological process | GO:0070271 | protein complex biogenesis | 0.046887733 | 30 | MIF/MSRB1/RACK1/TLR4/PET100/CLU/RPS3/PPP2R1A/RDX/CAPZA2/CDC42/COX17/PSMC5/MX2/COX3/HSD17B8/CD3D/SOX9/PRNP/Fbiological_process1/CAPN3/MET/VEGFA/FCER1G/SUMO1/TFAM/HMGB1/AIF1/SLC9A1/ESR1 |
| Biological process | GO:0034622 | cellular macromolecular complex assembly | 0.006883154 | 28 | RPS15/MSRB1/RPSA/TLR4/PET100/SRSF1/SNRPD2/RPS28/RPS3/RPL10/RDX/CAPZA2/RPL13A/COX17/PSMC5/MX2/COX3/HMGB2/RPS19/RPL3/RPL6/SOX9/MET/TFAM/HMGB1/DDX39B/AIF1/ESR1 |
| Biological process | GO:0015672 | monovalent inorganic cation transport | 0.03100601 | 26 | COX7A1/COX5B/ATP5D/ATP6V1A/ATP5G1/COX3/COX7C/ATP1B1/COX6C/ATP5J/ATP5I/SLC5A10/OXSR1/PRNP/ADRA2A/KCNH2/ATP8/UCP3/SUMO1/ATP6/SLC4A4/AQP1/SLC5A4/SLC9A1/SLC5A1/SLC35A4 |
| Biological process | GO:0006732 | coenzyme metabolic process | 0.000101273 | 25 | NAXE/OGT/GAPDH/PRKAA1/TALDO1/NAMPT/SUCLG2/FTCD/PDHA1/ENO3/PMVK/AHCY/QDPR/COASY/PDXK/Fbiological_process1/GNMT/FMO1/GPI/RPIA/TPI1/HMGCR/MOCS3/PFKM/VNN1 |
| Biological process | GO:0051186 | cofactor metabolic process | 0.000444501 | 25 | NAXE/OGT/GAPDH/PRKAA1/TALDO1/NAMPT/SUCLG2/FTCD/PDHA1/ENO3/PMVK/AHCY/QDPR/COASY/PDXK/Fbiological_process1/GNMT/FMO1/GPI/RPIA/TPI1/HMGCR/MOCS3/PFKM/VNN1 |
| Biological process | GO:0044723 | single-organism carbohydrate metabolic process | 0.004311903 | 24 | OGT/GAPDH/PRKAA1/MGAT2/IDH2/PDHA1/GULO/ENO3/IMPA1/IGF2/Fbiological_process1/UGP2/IMPAD1/GSTO1/MAN2B2/GPI/RPIA/GOT1/DHDH/TPI1/IGF1/PFKM/TKFC/NEU1 |
| Biological process | GO:0009894 | regulation of catabolic process | 0.006962409 | 24 | APOC3/RACK1/APOA1/OGT/CLU/PRKAA1/ARAF/TIMP1/CDC42/PSMC5/ITGB1/RNF114/PSME1/BAG6/RNF19A/LEPR/PSME2/ADRA2A/Fbiological_process1/APOA4/SUMO1/PRKAA2/SCARB1/CDK4 |
| Biological process | GO:0009123 | nucleoside monophosphate metabolic process | 0.047429715 | 23 | AK1/COX5B/OGT/GUK1/GAPDH/PRKAA1/ATP5D/ATP6V1A/ATP5G1/COX7C/ATP1B1/ENO3/PPARGC1A/ATP5J/ATP5I/ADSS/Fbiological_process1/ATP8/SDHA/GPI/ATP6/TPI1/PFKM |
| Biological process | GO:0009126 | purine nucleoside monophosphate metabolic process | 0.047429715 | 23 | AK1/COX5B/OGT/GUK1/GAPDH/PRKAA1/ATP5D/ATP6V1A/ATP5G1/COX7C/ATP1B1/ENO3/PPARGC1A/ATP5J/ATP5I/ADSS/Fbiological_process1/ATP8/SDHA/GPI/ATP6/TPI1/PFKM |
| Biological process | GO:0009161 | ribonucleoside monophosphate metabolic process | 0.047429715 | 23 | AK1/COX5B/OGT/GUK1/GAPDH/PRKAA1/ATP5D/ATP6V1A/ATP5G1/COX7C/ATP1B1/ENO3/PPARGC1A/ATP5J/ATP5I/ADSS/Fbiological_process1/ATP8/SDHA/GPI/ATP6/TPI1/PFKM |
| Biological process | GO:0009167 | purine ribonucleoside monophosphate metabolic process | 0.047429715 | 23 | AK1/COX5B/OGT/GUK1/GAPDH/PRKAA1/ATP5D/ATP6V1A/ATP5G1/COX7C/ATP1B1/ENO3/PPARGC1A/ATP5J/ATP5I/ADSS/Fbiological_process1/ATP8/SDHA/GPI/ATP6/TPI1/PFKM |
| Biological process | GO:0030163 | protein catabolic process | 0.047429715 | 23 | APOE/PSMB7/RACK1/PSMB4/CLU/CAST/ARAF/TIMP1/CDC42/PSMC5/UCHL1/RNF114/PSME1/BAG6/RNF19A/CTSB/CTSH/PSME2/ADRA2A/VCP/GNRHR/SUMO1/USP37 |
| Biological process | GO:0009144 | purine nucleoside triphosphate metabolic process | 0.035482201 | 22 | AK1/NME2/COX5B/OGT/GAPDH/PRKAA1/ATP5D/ATP6V1A/ATP5G1/COX7C/ATP1B1/ENO3/PPARGC1A/ATP5J/ATP5I/Fbiological_process1/ATP8/SDHA/GPI/ATP6/TPI1/PFKM |
| Biological process | GO:0009199 | ribonucleoside triphosphate metabolic process | 0.035482201 | 22 | AK1/NME2/COX5B/OGT/GAPDH/PRKAA1/ATP5D/ATP6V1A/ATP5G1/COX7C/ATP1B1/ENO3/PPARGC1A/ATP5J/ATP5I/Fbiological_process1/ATP8/SDHA/GPI/ATP6/TPI1/PFKM |
| Biological process | GO:0009205 | purine ribonucleoside triphosphate metabolic process | 0.035482201 | 22 | AK1/NME2/COX5B/OGT/GAPDH/PRKAA1/ATP5D/ATP6V1A/ATP5G1/COX7C/ATP1B1/ENO3/PPARGC1A/ATP5J/ATP5I/Fbiological_process1/ATP8/SDHA/GPI/ATP6/TPI1/PFKM |
| Biological process | GO:0009141 | nucleoside triphosphate metabolic process | 0.04928384 | 22 | AK1/NME2/COX5B/OGT/GAPDH/PRKAA1/ATP5D/ATP6V1A/ATP5G1/COX7C/ATP1B1/ENO3/PPARGC1A/ATP5J/ATP5I/Fbiological_process1/ATP8/SDHA/GPI/ATP6/TPI1/PFKM |
| Biological process | GO:0044282 | small molecule catabolic process | 0.010840296 | 21 | APOE/FAAH/ETFB/HPD/NPL/FTCD/UOX/IMPA1/AHCY/GPT/OXCT1/HADHA/RENbiological_process/GOT1/DHDH/CPT1B/ABAT/TPI1/TDH/SCARB1/ACADL |
| Biological process | GO:0006631 | fatty acid metabolic process | 0.036584082 | 21 | FAM213B/MIF/APOC3/APOA1/FAAH/ETFB/PRKAA1/PTGDS/CYP2D25/HSD17B8/CYP2E1/PPARGC1A/CYP2C42/HADHA/C3/APOA4/PRKAA2/CPT1B/CRYL1/ACADS/ACADL |
| Biological process | GO:0002253 | activation of immune response | 0.037657389 | 20 | PGLYRP2/LOC100513261/TLR4/NFKBIL1/RPS3/CFB/CFD/BAG6/RSAD2/PRNP/C3/FCER1G/C1S/HMGB1/CD59/TLR9/ESR1/TRAF6/TKFC/MB21D1 |
| Biological process | GO:1902532 | negative regulation of intracellular signal transduction | 0.000140903 | 19 | MIF/BGN/RACK1/HSPB1/CLU/PRKAA1/STAT1/IGF1R/LEPROT/SOCS2/UCHL1/PRNP/Fbiological_process1/MET/PRKAA2/DCN/BCL2L1/ESR1/TKFC |
| Biological process | GO:0070647 | protein modification by small protein conjugation or removal | 0.001768415 | 19 | RACK1/OGT/UBE2D3/COPS6/CLU/RPS3/CDC42/SOCS2/UIMC1/UCHL1/RNF114/RNF19A/CAPN3/VCP/GNRHR/SUMO1/USP37/MOCS3/TRAF6 |
| Biological process | GO:0031329 | regulation of cellular catabolic process | 0.026199074 | 19 | APOC3/RACK1/APOA1/CLU/PRKAA1/ARAF/TIMP1/PSMC5/RNF114/PSME1/BAG6/RNF19A/LEPR/PSME2/ADRA2A/APOA4/SUMO1/PRKAA2/SCARB1 |
| Biological process | GO:0006979 | response to oxidative stress | 0.038686219 | 19 | GPX1/GPX4/ROMO1/MSRB1/RACK1/HSPB1/RPS3/TXNIP/TPO/DPEP1/PPARGC1A/OXSR1/PRNP/APP/MET/HYAL1/ETFDH/AQP1/TXNRD1 |
| Biological process | GO:0051603 | proteolysis involved in cellular protein catabolic process | 0.016927429 | 18 | PSMB7/RACK1/PSMB4/CLU/ARAF/PSMC5/UCHL1/RNF114/PSME1/BAG6/RNF19A/CTSB/CTSH/PSME2/VCP/GNRHR/SUMO1/USP37 |
| Biological process | GO:0022613 | ribonucleoprotein complex biogenesis | 0.039650504 | 18 | RPS15/RPL7A/RPSA/RPL27/SRSF1/RPS21/SNRPD2/RPS28/RPS16/RPL10/RPLP0/RPL13A/RPS19/RPL3/RPL6/RPL35/DDX39B/ABCF1 |
| Biological process | GO:0044257 | cellular protein catabolic process | 0.039650504 | 18 | PSMB7/RACK1/PSMB4/CLU/ARAF/PSMC5/UCHL1/RNF114/PSME1/BAG6/RNF19A/CTSB/CTSH/PSME2/VCP/GNRHR/SUMO1/USP37 |
| Biological process | GO:0002764 | immune response-regulating signaling pathway | 0.009392923 | 16 | PGLYRP2/LOC100513261/KIT/TLR4/NFKBIL1/RPS3/BAG6/RSAD2/CTSH/PRNP/FCER1G/HMGB1/TLR9/ESR1/TRAF6/TKFC |
| Biological process | GO:0048871 | multicellular organismal homeostasis | 0.026723739 | 16 | ADRB1/CFTR/HSPB1/PRKAA1/HAMP/PTH1R/PPARGC1A/LEPR/CTSH/SOX9/MET/IL1B/SPP1/AQP1/TLR9/TRAF6 |
| Biological process | GO:0032446 | protein modification by small protein conjugation | 0.002030186 | 15 | RACK1/OGT/UBE2D3/CLU/RPS3/CDC42/SOCS2/RNF114/RNF19A/CAPN3/VCP/GNRHR/SUMO1/MOCS3/TRAF6 |
| Biological process | GO:0072524 | pyridine-containing compound metabolic process | 0.004448932 | 15 | NAXE/OGT/GAPDH/PRKAA1/TALDO1/NAMPT/ACMSD/ENO3/PDXK/Fbiological_process1/FMO1/GPI/RPIA/TPI1/PFKM |
| Biological process | GO:0006818 | hydrogen transport | 0.041864957 | 15 | COX7A1/COX5B/ATP5D/ATP6V1A/ATP5G1/COX3/COX7C/COX6C/ATP5J/ATP5I/ATP8/UCP3/ATP6/SLC9A1/SLC35A4 |
| Biological process | GO:0015992 | proton transport | 0.041864957 | 15 | COX7A1/COX5B/ATP5D/ATP6V1A/ATP5G1/COX3/COX7C/COX6C/ATP5J/ATP5I/ATP8/UCP3/ATP6/SLC9A1/SLC35A4 |
| Biological process | GO:0030258 | lipid modification | 0.003820271 | 14 | APOE/LCAT/APOA1/ETFB/GGTA1P/PIP4K2A/IMPA1/PPARGC1A/HADHA/APOA4/IMPAD1/PIK3C3/CPT1B/PIK3CG |
| Biological process | GO:0002757 | immune response-activating signal transduction | 0.015093543 | 14 | PGLYRP2/LOC100513261/TLR4/NFKBIL1/RPS3/BAG6/RSAD2/PRNP/FCER1G/HMGB1/TLR9/ESR1/TRAF6/TKFC |
| Biological process | GO:0016054 | organic acid catabolic process | 0.015093543 | 14 | FAAH/ETFB/HPD/NPL/FTCD/AHCY/GPT/HADHA/RENbiological_process/GOT1/CPT1B/ABAT/TDH/ACADL |
| Biological process | GO:0046395 | carboxylic acid catabolic process | 0.015093543 | 14 | FAAH/ETFB/HPD/NPL/FTCD/AHCY/GPT/HADHA/RENbiological_process/GOT1/CPT1B/ABAT/TDH/ACADL |
| Biological process | GO:0006733 | oxidoreduction coenzyme metabolic process | 0.014110964 | 13 | NAXE/OGT/GAPDH/PRKAA1/TALDO1/NAMPT/ENO3/Fbiological_process1/FMO1/GPI/RPIA/TPI1/PFKM |
| Biological process | GO:0019362 | pyridine nucleotide metabolic process | 0.014110964 | 13 | NAXE/OGT/GAPDH/PRKAA1/TALDO1/NAMPT/ENO3/Fbiological_process1/FMO1/GPI/RPIA/TPI1/PFKM |
| Biological process | GO:0046496 | nicotinamide nucleotide metabolic process | 0.014110964 | 13 | NAXE/OGT/GAPDH/PRKAA1/TALDO1/NAMPT/ENO3/Fbiological_process1/FMO1/GPI/RPIA/TPI1/PFKM |
| Biological process | GO:0005996 | monosaCellular componentharide metabolic process | 0.025465216 | 13 | OGT/GAPDH/PDHA1/GULO/IGF2/Fbiological_process1/GSTO1/MAN2B2/GPI/RPIA/DHDH/TPI1/IGF1 |
| Biological process | GO:1902600 | hydrogen ion transmembrane transport | 0.025465216 | 13 | COX7A1/COX5B/ATP5D/ATP6V1A/ATP5G1/COX3/COX7C/COX6C/ATP5J/ATP5I/ATP8/ATP6/SLC9A1 |
| Biological process | GO:0007596 | blood coagulation | 0.042319184 | 13 | HSPB1/MYL9/PROC/F2/F12/FGA/PLG/ADRA2A/FGB/RAP2B/FCER1G/VCL/PIK3CG |
| Biological process | GO:0007599 | hemostasis | 0.042319184 | 13 | HSPB1/MYL9/PROC/F2/F12/FGA/PLG/ADRA2A/FGB/RAP2B/FCER1G/VCL/PIK3CG |
| Biological process | GO:0050817 | coagulation | 0.042319184 | 13 | HSPB1/MYL9/PROC/F2/F12/FGA/PLG/ADRA2A/FGB/RAP2B/FCER1G/VCL/PIK3CG |
| Biological process | GO:0002181 | cytoplasmic translation | 0.000171659 | 12 | RPLP2/RPLP1/RPS20/RPL29/RPS3/RPL27A/RPL13A/RPL6/RPL31/RPL15/RPL4/RPL22 |
| Biological process | GO:0016567 | protein ubiquitination | 0.002372771 | 12 | RACK1/OGT/UBE2D3/CLU/RPS3/CDC42/SOCS2/RNF114/RNF19A/VCP/GNRHR/TRAF6 |
| Biological process | GO:0008643 | carbohydrate transport | 0.012844819 | 12 | SLC2A2/SLC2A3/SLC2A4/SLC5A10/C3/SLC35D1/AQP1/IGF1/SLC5A4/SLC5A1/EDNRA/SLC35A4 |
| Biological process | GO:0006511 | ubiquitin-dependent protein catabolic process | 0.02439453 | 12 | RACK1/CLU/ARAF/PSMC5/UCHL1/RNF114/BAG6/RNF19A/VCP/GNRHR/SUMO1/USP37 |
| Biological process | GO:0019941 | modification-dependent protein catabolic process | 0.02439453 | 12 | RACK1/CLU/ARAF/PSMC5/UCHL1/RNF114/BAG6/RNF19A/VCP/GNRHR/SUMO1/USP37 |
| Biological process | GO:0043632 | modification-dependent macromolecule catabolic process | 0.02439453 | 12 | RACK1/CLU/ARAF/PSMC5/UCHL1/RNF114/BAG6/RNF19A/VCP/GNRHR/SUMO1/USP37 |
| Biological process | GO:0051348 | negative regulation of transferase activity | 0.02439453 | 12 | LOC100513261/BGN/RACK1/GNAQ/WNK1/HSPB1/PRKAR2A/PKIG/IGF1R/SOCS2/UCHL1/DCN |
| Biological process | GO:0009132 | nucleoside diphosphate metabolic process | 0.011256669 | 11 | AK1/NME2/OGT/GUK1/GAPDH/PRKAA1/ENO3/Fbiological_process1/GPI/TPI1/PFKM |
| Biological process | GO:0046939 | nucleotide phosphorylation | 0.011256669 | 11 | AK1/NME2/OGT/GUK1/GAPDH/PRKAA1/ENO3/Fbiological_process1/GPI/TPI1/PFKM |
| Biological process | GO:0010498 | proteasomal protein catabolic process | 0.022861803 | 11 | RACK1/CLU/ARAF/PSMC5/RNF114/PSME1/BAG6/RNF19A/PSME2/VCP/SUMO1 |
| Biological process | GO:0046165 | alcohol biosynthetic process | 0.022861803 | 11 | APOE/APOA1/PRKAA1/IMPA1/PMVK/APOA4/IMPAD1/GOT1/PRKAA2/FGF1/HMGCR |
| Biological process | GO:0006469 | negative regulation of protein kinase activity | 0.041226274 | 11 | BGN/RACK1/GNAQ/WNK1/HSPB1/PRKAR2A/PKIG/IGF1R/SOCS2/UCHL1/DCN |
| Biological process | GO:0022618 | ribonucleoprotein complex assembly | 0.041226274 | 11 | RPS15/RPSA/SRSF1/SNRPD2/RPS28/RPL10/RPL13A/RPS19/RPL3/RPL6/DDX39B |
| Biological process | GO:0033673 | negative regulation of kinase activity | 0.041226274 | 11 | BGN/RACK1/GNAQ/WNK1/HSPB1/PRKAR2A/PKIG/IGF1R/SOCS2/UCHL1/DCN |
| Biological process | GO:0061136 | regulation of proteasomal protein catabolic process | 0.009318062 | 10 | RACK1/CLU/ARAF/PSMC5/RNF114/PSME1/BAG6/RNF19A/PSME2/SUMO1 |
| Biological process | GO:0006165 | nucleoside diphosphate phosphorylation | 0.020758689 | 10 | AK1/NME2/OGT/GAPDH/PRKAA1/ENO3/Fbiological_process1/GPI/TPI1/PFKM |
| Biological process | GO:0016051 | carbohydrate biosynthetic process | 0.020758689 | 10 | OGT/MGAT2/GULO/IMPA1/Fbiological_process1/IMPAD1/GPI/GOT1/TPI1/IGF1 |
| Biological process | GO:1903050 | regulation of proteolysis involved in cellular protein catabolic process | 0.020758689 | 10 | RACK1/CLU/ARAF/PSMC5/RNF114/PSME1/BAG6/RNF19A/PSME2/SUMO1 |
| Biological process | GO:0009142 | nucleoside triphosphate biosynthetic process | 0.03976273 | 10 | AK1/NME2/COX5B/ATP5D/ATP5G1/PPARGC1A/ATP5J/ATP5I/ATP8/ATP6 |
| Biological process | GO:0044724 | single-organism carbohydrate catabolic process | 0.03976273 | 10 | OGT/GAPDH/PRKAA1/ENO3/Fbiological_process1/GPI/DHDH/TPI1/PFKM/NEU1 |
| Biological process | GO:0045089 | positive regulation of innate immune response | 0.03976273 | 10 | PGLYRP2/TLR4/NFKBIL1/RSAD2/HMGB1/TLR9/ESR1/TKFC/KLRK1/MB21D1 |
| Biological process | GO:1903362 | regulation of cellular protein catabolic process | 0.03976273 | 10 | RACK1/CLU/ARAF/PSMC5/RNF114/PSME1/BAG6/RNF19A/PSME2/SUMO1 |
| Biological process | GO:0009135 | purine nucleoside diphosphate metabolic process | 0.017961324 | 9 | OGT/GUK1/GAPDH/PRKAA1/ENO3/Fbiological_process1/GPI/TPI1/PFKM |
| Biological process | GO:0009179 | purine ribonucleoside diphosphate metabolic process | 0.017961324 | 9 | OGT/GUK1/GAPDH/PRKAA1/ENO3/Fbiological_process1/GPI/TPI1/PFKM |
| Biological process | GO:0009185 | ribonucleoside diphosphate metabolic process | 0.017961324 | 9 | OGT/GUK1/GAPDH/PRKAA1/ENO3/Fbiological_process1/GPI/TPI1/PFKM |
| Biological process | GO:0031647 | regulation of protein stability | 0.017961324 | 9 | GNAQ/GAPDH/CLU/CDC42/ATP1B1/BAG6/CTSH/PRNP/SUMO1 |
| Biological process | GO:0006090 | pyruvate metabolic process | 0.037408666 | 9 | OGT/GAPDH/PRKAA1/PDHA1/ENO3/Fbiological_process1/GPI/TPI1/PFKM |
| Biological process | GO:0009145 | purine nucleoside triphosphate biosynthetic process | 0.037408666 | 9 | NME2/COX5B/ATP5D/ATP5G1/PPARGC1A/ATP5J/ATP5I/ATP8/ATP6 |
| Biological process | GO:0009201 | ribonucleoside triphosphate biosynthetic process | 0.037408666 | 9 | NME2/COX5B/ATP5D/ATP5G1/PPARGC1A/ATP5J/ATP5I/ATP8/ATP6 |
| Biological process | GO:0009206 | purine ribonucleoside triphosphate biosynthetic process | 0.037408666 | 9 | NME2/COX5B/ATP5D/ATP5G1/PPARGC1A/ATP5J/ATP5I/ATP8/ATP6 |
| Biological process | GO:0043161 | proteasome-mediated ubiquitin-dependent protein catabolic process | 0.037408666 | 9 | RACK1/CLU/ARAF/PSMC5/RNF114/BAG6/RNF19A/VCP/SUMO1 |
| Biological process | GO:0060560 | developmental growth involved in morphogenesis | 0.014347497 | 8 | APOE/SOX9/APP/FGF1/RAB11A/ESR1/NTN1/VCL |
| Biological process | GO:0000041 | transition metal ion transport | 0.033857412 | 8 | TFRC/FTL/SLC39A7/SLC31A1/COX17/HAMP/FTH1/TCN1 |
| Biological process | GO:0006096 | glycolytic process | 0.033857412 | 8 | OGT/GAPDH/PRKAA1/ENO3/Fbiological_process1/GPI/TPI1/PFKM |
| Biological process | GO:0006695 | cholesterol biosynthetic process | 0.033857412 | 8 | APOE/APOA1/PRKAA1/PMVK/APOA4/PRKAA2/FGF1/HMGCR |
| Biological process | GO:0006757 | ATP generation from ADP | 0.033857412 | 8 | OGT/GAPDH/PRKAA1/ENO3/Fbiological_process1/GPI/TPI1/PFKM |
| Biological process | GO:0046031 | ADP metabolic process | 0.033857412 | 8 | OGT/GAPDH/PRKAA1/ENO3/Fbiological_process1/GPI/TPI1/PFKM |
| Biological process | GO:1902653 | secondary alcohol biosynthetic process | 0.033857412 | 8 | APOE/APOA1/PRKAA1/PMVK/APOA4/PRKAA2/FGF1/HMGCR |
| Cellular component | GO:0044422 | organelle part | 0.032117155 | 194 | RPLP2/RPLP1/MIF/SNRPA/RPS15/COX7A1/RPS20/RPL7A/ROMO1/POLR2I/MTX1/RPSA/PSMB7/RACK1/GNAQ/COX5B/OGT/FAAH/ETFB/HPD/ND3/GGTA1P/CFTR/RPL29/HSPB1/PET100/RPL27/ERO1A/COPS6/SRSF1/RPS21/PRELID3B/SNRPD2/CLU/C2H19orf70/S100A6/KLF9/RPS28/DES/RPS9/DDOST/RPS16/PTGDS/CSNK2B/RPS3/MYL9/PPP2R1A/POR/ATP5D/STAT1/MRPS18B/RPL10/CYP2D25/ATP6V1A/ATP5G1/RPL27A/SLC39A7/FUNDC2/CAPZA2/MYL6/RPL13A/NR3C1/RPS29/DNAL4/RPL21/CDC42/RNH1/ADAM10/IYD/COX17/LMNA/RPN1/PSMC5/SREBF1/MX2/COX3/CFL1/TNNT1/SLC25A6/AR/HMGB2/MGAT2/RPS23/COX7C/FTCD/RPS19/LEPROT/HSD17B8/UIMC1/PDHA1/RPL3/GULO/RPL6/CYP2E1/DPEP1/CSNK1A1/U2AF1/UCHL1/JAK2/RPL35/CBR2/CAMK2D/TMEM14A/COX6C/CSTB/PPARGC1A/RAB34/DPP4/RPS12/RPL31/BAG6/VIM/RNF19A/ATP5J/ATP5I/RSAD2/NRAS/TMEM57/COASY/MYL4/CYP2C42/RPL15/MYO7A/OXCT1/PRNP/CLCN5/RAB14/HADHA/GATM/APP/RAP2B/CAPN3/CYP3A29/SLC35D1/VCP/ATP8/UCP3/RPL4/TSPO/RPL22/GALNT1/FMO1/IMPAD1/SUMO1/DAD1/TOP2A/SDHA/ITM2B/GOT1/SORD/PRKAA2/VAPB/NR5A1/SCAMP1/ATP6/NPC1/PLIN3/GPAM/CPT1B/NLN/TFAM/HMGB1/ETFDH/ABAT/RPS13/SUCLG1/BCL2L1/AQP1/DDX39B/BECN1/HMGCR/AIF1/RAB11A/TLR9/MAOA/FASLG/SCARB1/ABCF1/SLC9A1/B3GNT5/MUT/ACADS/ESR1/CDK4/ACADL/GUCA2B/VCL/FUT8/WT1/NEU1/MDH2/VDAC1/SLC35A4/RBM4B |
| Cellular component | GO:0044446 | intracellular organelle part | 0.025217557 | 192 | RPLP2/RPLP1/MIF/SNRPA/RPS15/COX7A1/RPS20/RPL7A/ROMO1/POLR2I/MTX1/RPSA/PSMB7/RACK1/GNAQ/COX5B/OGT/FAAH/ETFB/HPD/ND3/GGTA1P/CFTR/RPL29/HSPB1/PET100/RPL27/ERO1A/COPS6/SRSF1/RPS21/PRELID3B/SNRPD2/CLU/C2H19orf70/S100A6/KLF9/RPS28/DES/RPS9/DDOST/RPS16/PTGDS/CSNK2B/RPS3/MYL9/PPP2R1A/POR/ATP5D/STAT1/MRPS18B/RPL10/CYP2D25/ATP6V1A/ATP5G1/RPL27A/SLC39A7/FUNDC2/CAPZA2/MYL6/RPL13A/NR3C1/RPS29/DNAL4/RPL21/CDC42/RNH1/ADAM10/IYD/COX17/LMNA/RPN1/PSMC5/SREBF1/MX2/COX3/CFL1/TNNT1/SLC25A6/AR/HMGB2/MGAT2/RPS23/COX7C/FTCD/RPS19/LEPROT/HSD17B8/UIMC1/PDHA1/RPL3/GULO/RPL6/CYP2E1/DPEP1/CSNK1A1/U2AF1/UCHL1/JAK2/RPL35/CBR2/CAMK2D/TMEM14A/COX6C/CSTB/PPARGC1A/RAB34/DPP4/RPS12/RPL31/BAG6/VIM/RNF19A/ATP5J/ATP5I/RSAD2/NRAS/TMEM57/COASY/MYL4/CYP2C42/RPL15/MYO7A/OXCT1/PRNP/CLCN5/RAB14/HADHA/GATM/APP/RAP2B/CYP3A29/SLC35D1/VCP/ATP8/UCP3/RPL4/TSPO/RPL22/GALNT1/FMO1/IMPAD1/SUMO1/DAD1/TOP2A/SDHA/ITM2B/GOT1/SORD/PRKAA2/VAPB/NR5A1/SCAMP1/ATP6/NPC1/PLIN3/GPAM/CPT1B/NLN/TFAM/HMGB1/ETFDH/ABAT/RPS13/SUCLG1/BCL2L1/AQP1/DDX39B/BECN1/HMGCR/AIF1/RAB11A/TLR9/MAOA/FASLG/SCARB1/ABCF1/SLC9A1/B3GNT5/MUT/ACADS/ESR1/CDK4/ACADL/VCL/FUT8/WT1/NEU1/MDH2/VDAC1/SLC35A4/RBM4B |
| Cellular component | GO:0032991 | macromolecular complex | 0.001638425 | 138 | RPLP2/RPLP1/APOE/SNRPA/RPS15/RPS20/RPL7A/POLR2I/APOC3/RPSA/UBA52/FAU/PSMB7/RACK1/GNAQ/COX5B/FTL/APOA1/TLR4/OGT/ND3/CFTR/RPL29/RAMP1/RPL27/PSMB4/COPS6/SRSF1/GAPDH/RPS21/SNRPD2/DCPS/CLU/C2H19orf70/PRKAA1/RPS28/RPS9/DDOST/RPS16/CSNK2B/RPS3/MYL9/ATP5D/MRPS18B/RPL10/ATP6V1A/ATP5G1/RPL27A/RPLP0/CAPZA2/MYL6/RPL13A/RABGGTA/RPS29/DNAL4/RPL21/CDC42/RNH1/GP91-PHOX/RPN1/GNAS/PSMC5/MX2/COX3/TNNT1/RPS23/COX7C/ATP1B1/SLA-DQB1/RPS19/UIMC1/PDHA1/RPL3/ITGB1/RPL6/CASP1/CSNK1A1/U2AF1/APOM/RPL35/ENO3/-/CD3D/RPS12/RPL31/PSME1/BAG6/RNF19A/ATP5J/ATP5I/MYL4/RPL15/MYO7A/CTSH/SOX9/CYBA/PSME2/Mbiological_process/PRNP/ADRA2A/HADHA/APP/CAPN3/EIF2S3/ATP8/RTCB/RPL4/RPL22/APOA4/FCER1G/ATM/SUMO1/PIK3C3/DAD1/TOP2A/SDHA/GON7/PRKAA2/NR5A1/PRKAR1A/ATP6/ETFDH/ABAT/RPS13/DDX39B/SFTPA1/BECN1/IGF1/RAB11A/ABCF1/ESR1/CDK4/TRAF6/VCL/PIK3CG/FCN1/VDAC1/RBM4B |
| Cellular component | GO:0005829 | cytosol | 0.000267366 | 89 | RPLP2/RPLP1/FAM213B/MIF/LOC100513261/RPS15/RPS20/RPL7A/RPSA/PSMB7/RACK1/OGT/GUK1/ETFB/CFTR/RPL29/RPL27/GAPDH/RPS21/SNRPD2/CLU/S100A6/KLF9/RPS28/RPS9/RPS16/RPS3/RPL10/ATP6V1A/RPL27A/ARAF/RPL13A/RPS29/RPL21/CDC42/RNH1/PSMC5/RPS23/RPS19/PPIA/RPL3/RPL6/CASP1/ACMSD/UCHL1/RPL35/ENO3/PMVK/CSTB/AHCY/RPS12/RPL31/BAG6/ADSS/RPL15/OXSR1/CTSH/PRNP/PDXK/RAB14/SAT1/Fbiological_process1/RAP2B/CAPN3/AFP/VCP/RPL4/RPL22/ASPA/IMPAD1/PIK3C3/GSTO1/RPIA/GOT1/IL1B/NLN/ABAT/TPI1/CRYL1/RPS13/BCL2L1/FGF1/BECN1/AIF1/MOCS3/CDK4/TRAF6/MB21D1/RBM4B |
| Cellular component | GO:0005739 | mitochondrion | 0.013141115 | 72 | NAXE/COX7A1/GPX4/IDH3G/ROMO1/AKAP10/MTX1/RACK1/COX5B/ETFB/ND3/PET100/PRELID3B/CLU/C2H19orf70/RPS3/ATP5D/MRPS18B/ATP5G1/ARAF/FUNDC2/NR3C1/IDH3A/COX17/MX2/COX3/SUCLG2/TBRG4/SLC25A6/COX7C/HSD17B8/IDH2/PDHA1/DHRS4/CBR2/TMEM14A/COX6C/PRKACA/ATP5J/ATP5I/COASY/OXCT1/ACO2/HADHA/GATM/ATP8/UCP3/TSPO/FAS/SDHA/GOT1/SORD/ATP6/GPAM/CPT1B/NLN/TFAM/ETFDH/ABAT/SUCLG1/BCL2L1/BECN1/TDH/RAB11A/MAOA/MUT/ACADS/ACADL/TXNRD1/BCAT2/MDH2/VDAC1 |
| Cellular component | GO:0031974 | membrane-enclosed lumen | 0.038081366 | 63 | MIF/SNRPA/POLR2I/PSMB7/RACK1/OGT/ETFB/SRSF1/PRELID3B/KLF9/RPS3/STAT1/MRPS18B/NR3C1/RNH1/COX17/LMNA/PSMC5/CFL1/AR/HMGB2/HSD17B8/PDHA1/RPL3/CSNK1A1/U2AF1/UCHL1/JAK2/CBR2/CSTB/PPARGC1A/BAG6/COASY/OXCT1/HADHA/GATM/APP/IMPAD1/SUMO1/TOP2A/SDHA/GOT1/PRKAA2/NLN/TFAM/HMGB1/ETFDH/ABAT/RPS13/SUCLG1/BCL2L1/DDX39B/FASLG/ABCF1/MUT/ACADS/ESR1/CDK4/ACADL/WT1/NEU1/MDH2/RBM4B |
| Cellular component | GO:0043233 | organelle lumen | 0.038081366 | 63 | MIF/SNRPA/POLR2I/PSMB7/RACK1/OGT/ETFB/SRSF1/PRELID3B/KLF9/RPS3/STAT1/MRPS18B/NR3C1/RNH1/COX17/LMNA/PSMC5/CFL1/AR/HMGB2/HSD17B8/PDHA1/RPL3/CSNK1A1/U2AF1/UCHL1/JAK2/CBR2/CSTB/PPARGC1A/BAG6/COASY/OXCT1/HADHA/GATM/APP/IMPAD1/SUMO1/TOP2A/SDHA/GOT1/PRKAA2/NLN/TFAM/HMGB1/ETFDH/ABAT/RPS13/SUCLG1/BCL2L1/DDX39B/FASLG/ABCF1/MUT/ACADS/ESR1/CDK4/ACADL/WT1/NEU1/MDH2/RBM4B |
| Cellular component | GO:0070013 | intracellular organelle lumen | 0.038081366 | 63 | MIF/SNRPA/POLR2I/PSMB7/RACK1/OGT/ETFB/SRSF1/PRELID3B/KLF9/RPS3/STAT1/MRPS18B/NR3C1/RNH1/COX17/LMNA/PSMC5/CFL1/AR/HMGB2/HSD17B8/PDHA1/RPL3/CSNK1A1/U2AF1/UCHL1/JAK2/CBR2/CSTB/PPARGC1A/BAG6/COASY/OXCT1/HADHA/GATM/APP/IMPAD1/SUMO1/TOP2A/SDHA/GOT1/PRKAA2/NLN/TFAM/HMGB1/ETFDH/ABAT/RPS13/SUCLG1/BCL2L1/DDX39B/FASLG/ABCF1/MUT/ACADS/ESR1/CDK4/ACADL/WT1/NEU1/MDH2/RBM4B |
| Cellular component | GO:0044428 | nuclear part | 0.032179888 | 56 | MIF/SNRPA/POLR2I/PSMB7/RACK1/GNAQ/OGT/COPS6/SRSF1/SNRPD2/S100A6/KLF9/PTGDS/CSNK2B/RPS3/STAT1/MRPS18B/NR3C1/RNH1/LMNA/PSMC5/MX2/CFL1/AR/HMGB2/UIMC1/RPL3/CSNK1A1/U2AF1/UCHL1/JAK2/CSTB/PPARGC1A/BAG6/TMEM57/OXCT1/PRNP/APP/IMPAD1/SUMO1/TOP2A/SDHA/GOT1/PRKAA2/NR5A1/NPC1/HMGB1/RPS13/BCL2L1/AQP1/DDX39B/ABCF1/ESR1/CDK4/WT1/RBM4B |
| Cellular component | GO:0044429 | mitochondrial part | 0.042389465 | 51 | COX7A1/ROMO1/MTX1/COX5B/ETFB/ND3/PET100/PRELID3B/CLU/C2H19orf70/RPS3/ATP5D/MRPS18B/ATP5G1/FUNDC2/COX17/MX2/COX3/SLC25A6/COX7C/HSD17B8/PDHA1/CBR2/TMEM14A/COX6C/ATP5J/ATP5I/COASY/HADHA/GATM/ATP8/UCP3/TSPO/SDHA/SORD/ATP6/GPAM/CPT1B/NLN/TFAM/ETFDH/ABAT/SUCLG1/BCL2L1/BECN1/MAOA/MUT/ACADS/ACADL/MDH2/VDAC1 |
| Cellular component | GO:0031967 | organelle envelope | 0.044784782 | 50 | COX7A1/ROMO1/MTX1/GNAQ/COX5B/ND3/PET100/PRELID3B/CLU/C2H19orf70/S100A6/PTGDS/RPS3/ATP5D/ATP5G1/FUNDC2/COX17/LMNA/MX2/COX3/SLC25A6/COX7C/TMEM14A/COX6C/ATP5J/ATP5I/TMEM57/PRNP/HADHA/GATM/APP/ATP8/UCP3/TSPO/SUMO1/SDHA/SORD/ATP6/NPC1/GPAM/CPT1B/NLN/ETFDH/BCL2L1/AQP1/BECN1/MAOA/ABCF1/CDK4/VDAC1 |
| Cellular component | GO:0031975 | envelope | 0.044784782 | 50 | COX7A1/ROMO1/MTX1/GNAQ/COX5B/ND3/PET100/PRELID3B/CLU/C2H19orf70/S100A6/PTGDS/RPS3/ATP5D/ATP5G1/FUNDC2/COX17/LMNA/MX2/COX3/SLC25A6/COX7C/TMEM14A/COX6C/ATP5J/ATP5I/TMEM57/PRNP/HADHA/GATM/APP/ATP8/UCP3/TSPO/SUMO1/SDHA/SORD/ATP6/NPC1/GPAM/CPT1B/NLN/ETFDH/BCL2L1/AQP1/BECN1/MAOA/ABCF1/CDK4/VDAC1 |
| Cellular component | GO:0043230 | extracellular organelle | 0.027053183 | 48 | MIF/LOC100513261/APOC3/PSMB7/RACK1/GNAQ/COX5B/ETFB/HSPB1/RPS28/RPS16/RPS3/CAPZA2/MYL6/CDC42/RNH1/ATP1B1/PTH1R/UCHL1/PMVK/CSTB/DPP4/BAG6/IGFbiological_process2/COASY/ADSS/OXSR1/CTSH/PRNP/S100A11/GATM/Fbiological_process1/APP/RAP2B/ASPA/DAD1/ITM2B/GOT1/IL1B/SPP1/ARG1/ABAT/RAB11A/SCARB1/SAT2/GUCA2B/VCL/TKFC |
| Cellular component | GO:0070062 | extracellular exosome | 0.027053183 | 48 | MIF/LOC100513261/APOC3/PSMB7/RACK1/GNAQ/COX5B/ETFB/HSPB1/RPS28/RPS16/RPS3/CAPZA2/MYL6/CDC42/RNH1/ATP1B1/PTH1R/UCHL1/PMVK/CSTB/DPP4/BAG6/IGFbiological_process2/COASY/ADSS/OXSR1/CTSH/PRNP/S100A11/GATM/Fbiological_process1/APP/RAP2B/ASPA/DAD1/ITM2B/GOT1/IL1B/SPP1/ARG1/ABAT/RAB11A/SCARB1/SAT2/GUCA2B/VCL/TKFC |
| Cellular component | GO:1903561 | extracellular vesicle | 0.027053183 | 48 | MIF/LOC100513261/APOC3/PSMB7/RACK1/GNAQ/COX5B/ETFB/HSPB1/RPS28/RPS16/RPS3/CAPZA2/MYL6/CDC42/RNH1/ATP1B1/PTH1R/UCHL1/PMVK/CSTB/DPP4/BAG6/IGFbiological_process2/COASY/ADSS/OXSR1/CTSH/PRNP/S100A11/GATM/Fbiological_process1/APP/RAP2B/ASPA/DAD1/ITM2B/GOT1/IL1B/SPP1/ARG1/ABAT/RAB11A/SCARB1/SAT2/GUCA2B/VCL/TKFC |
| Cellular component | GO:1990904 | ribonucleoprotein complex | 4.48634E-06 | 47 | RPLP2/RPLP1/SNRPA/RPS15/RPS20/RPL7A/RPSA/UBA52/FAU/RACK1/RPL29/RPL27/SRSF1/RPS21/SNRPD2/DCPS/RPS28/RPS9/RPS16/RPS3/MRPS18B/RPL10/RPL27A/RPLP0/RPL13A/RPS29/RPL21/CDC42/RPS23/RPS19/RPL3/RPL6/U2AF1/RPL35/-/RPS12/RPL31/RPL15/CTSH/RPL4/RPL22/ATM/SUMO1/TOP2A/RPS13/DDX39B/ABCF1 |
| Cellular component | GO:0030529 | intracellular ribonucleoprotein complex | 7.77895E-06 | 46 | RPLP2/RPLP1/SNRPA/RPS15/RPS20/RPL7A/RPSA/UBA52/FAU/RACK1/RPL29/RPL27/SRSF1/RPS21/SNRPD2/DCPS/RPS28/RPS9/RPS16/RPS3/MRPS18B/RPL10/RPL27A/RPLP0/RPL13A/RPS29/RPL21/CDC42/RPS23/RPS19/RPL3/RPL6/U2AF1/RPL35/RPS12/RPL31/RPL15/CTSH/RPL4/RPL22/ATM/SUMO1/TOP2A/RPS13/DDX39B/ABCF1 |
| Cellular component | GO:0005840 | ribosome | 1.74029E-06 | 37 | RPLP2/RPLP1/RPS15/RPS20/RPL7A/RPSA/UBA52/FAU/RACK1/RPL29/RPL27/RPS21/RPS28/RPS9/RPS16/RPS3/MRPS18B/RPL10/RPL27A/RPLP0/RPL13A/RPS29/RPL21/RPS23/RPS19/RPL3/RPL6/RPL35/-/RPS12/RPL31/RPL15/RPL4/RPL22/ATM/RPS13/ABCF1 |
| Cellular component | GO:0044445 | cytosolic part | 0.000262559 | 33 | RPLP2/RPLP1/RPS15/RPS20/RPL7A/RPSA/RPL29/RPL27/RPS21/RPS28/RPS9/RPS16/RPS3/RPL10/RPL27A/RPL13A/RPS29/RPL21/PSMC5/RPS23/RPS19/RPL3/RPL6/CASP1/RPL35/ENO3/RPS12/RPL31/BAG6/RPL15/RPL4/RPL22/RPS13 |
| Cellular component | GO:0044391 | ribosomal subunit | 3.00736E-05 | 30 | RPLP2/RPLP1/RPS15/RPS20/RPL7A/RPSA/RPL29/RPL27/RPS21/RPS28/RPS9/RPS16/RPS3/MRPS18B/RPL10/RPL27A/RPL13A/RPS29/RPL21/RPS23/RPS19/RPL3/RPL6/RPL35/RPS12/RPL31/RPL15/RPL4/RPL22/RPS13 |
| Cellular component | GO:0022626 | cytosolic ribosome | 5.55629E-05 | 29 | RPLP2/RPLP1/RPS15/RPS20/RPL7A/RPSA/RPL29/RPL27/RPS21/RPS28/RPS9/RPS16/RPS3/RPL10/RPL27A/RPL13A/RPS29/RPL21/RPS23/RPS19/RPL3/RPL6/RPL35/RPS12/RPL31/RPL15/RPL4/RPL22/RPS13 |
| Cellular component | GO:0005743 | mitochondrial inner membrane | 0.038660148 | 22 | COX7A1/ROMO1/COX5B/ND3/PET100/C2H19orf70/RPS3/ATP5D/ATP5G1/COX3/SLC25A6/COX7C/COX6C/ATP5J/ATP5I/HADHA/GATM/ATP8/UCP3/SDHA/ATP6/ETFDH |
| Cellular component | GO:0005759 | mitochondrial matrix | 0.048096006 | 17 | ETFB/RPS3/MRPS18B/HSD17B8/PDHA1/CBR2/COASY/HADHA/TFAM/ETFDH/ABAT/SUCLG1/BCL2L1/MUT/ACADS/ACADL/MDH2 |
| Cellular component | GO:0015934 | large ribosomal subunit | 0.013011687 | 16 | RPLP2/RPLP1/RPL7A/RPL29/RPL27/RPL10/RPL27A/RPL13A/RPL21/RPL3/RPL6/RPL35/RPL31/RPL15/RPL4/RPL22 |
| Cellular component | GO:0022625 | cytosolic large ribosomal subunit | 0.013011687 | 16 | RPLP2/RPLP1/RPL7A/RPL29/RPL27/RPL10/RPL27A/RPL13A/RPL21/RPL3/RPL6/RPL35/RPL31/RPL15/RPL4/RPL22 |
| Cellular component | GO:1990234 | transferase complex | 0.034382311 | 15 | POLR2I/OGT/SNRPD2/PRKAA1/DDOST/RABGGTA/RPN1/RNF19A/PIK3C3/DAD1/PRKAA2/ABAT/BECN1/CDK4/PIK3CG |
| Cellular component | GO:0015935 | small ribosomal subunit | 0.000438305 | 14 | RPS15/RPS20/RPSA/RPS21/RPS28/RPS9/RPS16/RPS3/MRPS18B/RPS29/RPS23/RPS19/RPS12/RPS13 |
| Cellular component | GO:0022627 | cytosolic small ribosomal subunit | 0.000920127 | 13 | RPS15/RPS20/RPSA/RPS21/RPS28/RPS9/RPS16/RPS3/RPS29/RPS23/RPS19/RPS12/RPS13 |
| Molecular function | GO:0003824 | catalytic activity | 0.001557436 | 216 | HPX/PGLYRP2/NAXE/GPX1/AK1/FAM213B/MIF/LOC100513261/COX7A1/NME2/GPX4/IDH3G/POLR2I/Cellular componentS/MSRB1/KIT/PSMB7/GNAQ/COX5B/LCAT/WNK1/OGT/FAAH/GUK1/UBE2D3/PRDX2/HPD/ND3/GGTA1P/CFTR/PSMB4/ERO1A/TGFBR2/GAPDH/DCPS/CLU/PRKAA1/TALDO1/CAPN7/DDOST/NPL/PTGDS/RPS3/POR/AKR1A1/ATP5D/PROC/F2/CAPNS1/CYP2D25/ATP6V1A/ATP5G1/F12/ARAF/EEF1G/GMPPA/ACY1/MYL6/TMEM86B/RABGGTA/TPO/CFB/DNAL4/HEXB/CDC42/IDH3A/CA11/GP91-PHOX/ADAM10/IYD/RPN1/PIP4K2A/GNAS/NAMPT/PSMC5/MX2/COX3/SUCLG2/TBRG4/IGF1R/MGAT2/COX7C/FTCD/ATP1B1/HSD17B8/PPIA/PLAU/IDH2/PDHA1/GULO/CYP2E1/CASP1/DPEP1/CSNK1A1/ACMSD/UCHL1/JAK2/TGFBR3/EPX/RNF114/DHRS4/UOX/LDHA/ENO3/CBR2/CAMK2D/IMPA1/COX6C/CFD/PMVK/RAB34/DPP4/AHCY/PRKACA/PLPP1/PLG/CDKN3/RNF19A/DPYD/QDPR/ATP5I/RSAD2/CTSB/NRAS/ADAM17/GPT/COASY/CYP2C42/ADSS/MYO7A/OXSR1/OXCT1/CTSH/DIO2/SOX9/CYBA/PDXK/ACO2/FTH1/COMT/RAB14/SAT1/HADHA/GATM/DNASE1L1/Fbiological_process1/RAP2B/CAPN3/MET/GNMT/CASP3/HYAL1/CYP3A29/UGP2/GPATCH3/RENbiological_process/VCP/RTCB/GNRHR/GALNT1/ASPA/ATM/FMO1/IMPAD1/PIK3C3/DAD1/TOP2A/GSTO1/MAN2B2/ATP2B1/C1S/SDHA/USP37/GPI/RPIA/GOT1/SORD/PRKAA2/DHDH/GPAM/CA3/CPT1B/NLN/ARG1/MYLK/ETFDH/ABAT/TPI1/CRYL1/SUCLG1/DDX39B/TDH/HMGCR/DESI2/RAB11A/MAOA/MOCS3/ABCF1/B3GNT5/MUT/ACADS/CDK4/ACADL/SAT2/TRAF6/TXNRD1/PFKM/TKFC/PIK3CG/FUT8/ENTPD1/BCAT2/VNN1/NEU1/MDH2/MB21D1 |
| Molecular function | GO:0016740 | transferase activity | 0.000184053 | 70 | AK1/NME2/POLR2I/KIT/LCAT/WNK1/OGT/GUK1/UBE2D3/GGTA1P/TGFBR2/GAPDH/PRKAA1/TALDO1/DDOST/ARAF/EEF1G/GMPPA/RABGGTA/CDC42/RPN1/PIP4K2A/NAMPT/TBRG4/IGF1R/MGAT2/FTCD/CSNK1A1/JAK2/TGFBR3/RNF114/CAMK2D/PMVK/PRKACA/RNF19A/GPT/COASY/OXSR1/OXCT1/SOX9/PDXK/COMT/SAT1/GATM/MET/GNMT/HYAL1/UGP2/GALNT1/ATM/PIK3C3/DAD1/GSTO1/GOT1/PRKAA2/GPAM/CPT1B/MYLK/ABAT/MOCS3/B3GNT5/CDK4/SAT2/TRAF6/PFKM/TKFC/PIK3CG/FUT8/BCAT2/MB21D1 |
| Molecular function | GO:0098772 | molecular function regulator | 0.027794617 | 49 | RPLP1/APOE/LOC100513261/AMbiological_process/SERPINA1/BGN/Cellular componentS/APOC3/RACK1/GNAQ/APOA1/WNK1/ADRB1/GDI2/CFTR/HSPB1/PKIG/PPP1R14A/CSNK2B/PPP2R1A/CAST/TXNIP/TIMP1/PPP1R14B/RNH1/ATP1B1/SOCS2/RSC1A1/DPEP1/CSTB/ITIH1/PSME1/NGF/CTSH/PSME2/C3/APP/CAPN3/VEGFA/RENbiological_process/APOA4/SUMO1/ENSA/LOC396905/DCN/PRKAR1A/ITIH4/GUCA2B/SERPINA6 |
| Molecular function | GO:0030234 | enzyme regulator activity | 0.047552302 | 43 | RPLP1/APOE/AMbiological_process/SERPINA1/BGN/Cellular componentS/APOC3/RACK1/GNAQ/APOA1/WNK1/GDI2/HSPB1/PKIG/PPP1R14A/CSNK2B/PPP2R1A/CAST/TXNIP/TIMP1/PPP1R14B/RNH1/ATP1B1/SOCS2/DPEP1/CSTB/ITIH1/PSME1/NGF/CTSH/PSME2/C3/APP/CAPN3/RENbiological_process/APOA4/ENSA/LOC396905/DCN/PRKAR1A/ITIH4/GUCA2B/SERPINA6 |
| Molecular function | GO:0005198 | structural molecule activity | 0.001647771 | 42 | RPLP2/RPLP1/RPS15/RPS20/RPSA/UBA52/FAU/RPL29/RPL27/RPS21/RPS28/DES/RPS9/RPS16/RPS3/MRPS18B/RPL10/RPL27A/MYL6/RPL13A/RPS29/RPL21/LMNA/ACAN/RPS23/RPS19/RPL3/RPL6/RPL35/RPS12/RPL31/VIM/RPL15/Mbiological_process/CAPN3/RPL4/RPL22/ATM/FBN1/RPS13/CRYBB1/VCL |
| Molecular function | GO:0016772 | transferase activity, transferring phosphorus-containing groups | 0.006245315 | 35 | AK1/NME2/POLR2I/KIT/WNK1/GUK1/TGFBR2/PRKAA1/ARAF/GMPPA/PIP4K2A/TBRG4/IGF1R/CSNK1A1/JAK2/TGFBR3/CAMK2D/PMVK/PRKACA/COASY/OXSR1/SOX9/PDXK/MET/UGP2/ATM/PIK3C3/PRKAA2/MYLK/MOCS3/CDK4/PFKM/TKFC/PIK3CG/MB21D1 |
| Molecular function | GO:0003735 | structural constituent of ribosome | 4.10553E-06 | 32 | RPLP2/RPLP1/RPS15/RPS20/RPSA/UBA52/FAU/RPL29/RPL27/RPS21/RPS28/RPS9/RPS16/RPS3/MRPS18B/RPL10/RPL27A/RPL13A/RPS29/RPL21/RPS23/RPS19/RPL3/RPL6/RPL35/RPS12/RPL31/RPL15/RPL4/RPL22/ATM/RPS13 |
| Molecular function | GO:0016301 | kinase activity | 0.020744601 | 30 | AK1/NME2/KIT/WNK1/GUK1/TGFBR2/PRKAA1/ARAF/PIP4K2A/TBRG4/IGF1R/CSNK1A1/JAK2/TGFBR3/CAMK2D/PMVK/PRKACA/COASY/OXSR1/SOX9/PDXK/MET/ATM/PIK3C3/PRKAA2/MYLK/CDK4/PFKM/TKFC/PIK3CG |
| Molecular function | GO:0016773 | phosphotransferase activity, alcohol group as aCellular componenteptor | 0.005271487 | 27 | NME2/KIT/WNK1/TGFBR2/PRKAA1/ARAF/PIP4K2A/TBRG4/IGF1R/CSNK1A1/JAK2/TGFBR3/CAMK2D/PRKACA/COASY/OXSR1/SOX9/PDXK/MET/ATM/PIK3C3/PRKAA2/MYLK/CDK4/PFKM/TKFC/PIK3CG |
| Molecular function | GO:0004672 | protein kinase activity | 0.030145746 | 21 | NME2/KIT/WNK1/TGFBR2/PRKAA1/ARAF/TBRG4/IGF1R/CSNK1A1/JAK2/TGFBR3/CAMK2D/PRKACA/OXSR1/SOX9/MET/ATM/PRKAA2/MYLK/CDK4/PIK3CG |
| Molecular function | GO:0015077 | monovalent inorganic cation transmembrane transporter activity | 0.030145746 | 21 | COX7A1/COX5B/ATP5D/ATP6V1A/ATP5G1/COX3/COX7C/ATP1B1/COX6C/SLC28A1/ATP5J/ATP5I/SLC5A10/KCNH2/ATP8/ATP6/SLC4A4/AQP1/SLC5A4/SLC9A1/SLC5A1 |
| Molecular function | GO:0016614 | oxidoreductase activity, acting on CH-OH group of donors | 0.033328294 | 18 | FAM213B/IDH3G/AKR1A1/IDH3A/HSD17B8/IDH2/GULO/DHRS4/LDHA/CBR2/HADHA/SORD/DHDH/CRYL1/TDH/HMGCR/ENTPD1/MDH2 |
| Molecular function | GO:0015078 | hydrogen ion transmembrane transporter activity | 0.021925665 | 13 | COX7A1/COX5B/ATP5D/ATP6V1A/ATP5G1/COX3/COX7C/COX6C/ATP5J/ATP5I/ATP8/ATP6/SLC9A1 |
| Molecular function | GO:0016853 | isomerase activity | 0.019958448 | 11 | NAXE/MIF/ERO1A/PTGDS/PPIA/RENbiological_process/TOP2A/GPI/RPIA/TPI1/MUT |
| Molecular function | GO:0019887 | protein kinase regulator activity | 0.008114856 | 10 | RPLP1/BGN/RACK1/WNK1/HSPB1/PKIG/CSNK2B/SOCS2/DCN/PRKAR1A |
| Molecular function | GO:0019207 | kinase regulator activity | 0.018250341 | 10 | RPLP1/BGN/RACK1/WNK1/HSPB1/PKIG/CSNK2B/SOCS2/DCN/PRKAR1A |
| Molecular function | GO:0016758 | transferase activity, transferring hexosyl groups | 0.035283295 | 10 | OGT/GGTA1P/DDOST/RPN1/MGAT2/HYAL1/GALNT1/DAD1/B3GNT5/FUT8 |
