# Supplementary material for: Differentially Expressed Hepatic Genes Revealed by Transcriptomics in Pigs with Different Liver Lipid Contents
Source: Oxid Med Cell Longev. 2022 Jan 28;2022:2315575. doi: 10.1155/2022/2315575 (PMC8817107; doi:10.1155/2022/2315575)
Supplement: Supplementary 2 — Table S2: all enrichment pathways in KEGG analysis. [file 2315575.f2.docx]

| KEGGID | Description | P-value | Gene Number | Gene Name |
| --- | --- | --- | --- | --- |
| ssc03010 | Ribosome | 1.79557E-13 | 89 | RPS5/RPL28/RPLP2/RPLP1/RPL18A/RPS15/RPL12/RPS20/MRPL28/RPL23A/MRPS18A/MRPS15/RPL13/RPL8/MRPL24/RPS2/RPSA/UBA52/FAU/MRPL2/MRPL27/MRPL11/RPS11/RPL29/MRPL14/RPL27/RPL36/MRPS2/RPL19/LOC100518848/RPS21/RPS28/RN18S/RPS9/RPS16/MRPS11/RPS10/RPS3/MRPL12/RPL10/RPL27A/RPLP0/RN5-8S/RPS8/MRPL20/RPL13A/RPL38/RPS29/RPL39/MRPL36/RPL21/MRPL17/RPS23/RPS19/MRPL4/RPL3/MRPL3/RPL6/RPL35/RPL7/RPL37/RPS6/MRPL18/RPS12/RPL31/MRPL13/RPS27/MRPS21/RPS7/RPL26L1/RPL15/MRPS10/MRPS16/RPL24/RPL4/RPL22/MRPL16/MRPL10/RPL17/MRPS7/MRPL34/RPL22L1/LOC100623540/RPS13/RPS27A/RPL3L/MRPL32/MRPL35/RPS4X |
| ssc05010 | Alzheimer disease | 3.39854E-06 | 90 | NDUFB7/NDUFA11/NDUFA10/APOE/LOC100522725/LOC100624067/PSENEN/COX7A1/NDUFS8/NDUFV1/NDUFS3/NDUFB11/NDUFS7/GNAQ/COX5B/NDUFV3/HSD17B10/NDUFA3/NDUFA13/NDUFB9/FADD/GAPDH/COX6B/NDUFA8/ATP5D/NDUFC2/CASP8/LOC100525869/ATP5G1/MAPK3/RTN4/NDUFB10/NDUFA2/NDUFB2/LOC100516480/LOC100156879/RTN3/NDUFA7/ADAM10/ATP5G2/LOC100524613/NDUFS6/COX3/ATF6/CASP9/COX7C/NDUFA4/PPP3CB/NDUFB4/UQCR10/CDK5/APAF1/COX6C/NDUFA6/PPP3R1/NDUFB8/NDUFS5/ATP5J/ITPR1/BID/ADAM17/BAD/ITPR2/ATP5E/IDE/APBB1/COX6A1/APP/PSEN1/NDUFB1/CASP3/ATP8/ATP5G3/FAS/LOC100156967/NDUFV2/SDHA/NDUFS1/IL1B/ATP6/NDUFA1/LOC110257420/MAPK1/LOC100524308/MAPT/APH1A/NDUFAB1/LOC100524873/CYTB/CACNA1D |
| ssc04932 | Non-alcoholic fatty liver disease (NAFLD) | 1.55503E-05 | 80 | PPARA/NDUFB7/NDUFA11/NDUFA10/LOC100522725/LOC100624067/COX7A1/NDUFS8/NDUFV1/NDUFS3/NDUFB11/NDUFS7/COX5B/NDUFV3/NDUFA3/NDUFA13/NDUFB9/PRKAA1/COX6B/NDUFA8/NDUFC2/CASP8/LOC100525869/NDUFB10/NDUFA2/NDUFB2/LOC100516480/LOC100156879/NDUFA7/CDC42/SREBF1/LOC100524613/NDUFS6/COX3/PIK3R2/COX7C/NDUFA4/NDUFB4/CYP2E1/UQCR10/IL6R/COX6C/NDUFA6/AKT2/NDUFB8/PIK3CD/NDUFS5/MAPK9/NFKB1/LEPR/TRAF2/BID/CEBPA/DDIT3/NR1H3/COX6A1/NDUFB1/CASP3/GSK3A/FAS/LOC100156967/NDUFV2/MLXIP/SDHA/PRKAG2/NDUFS1/IL1B/PRKAA2/NDUFA1/LOC110257420/ADIPOR1/LOC100524308/ADIPOR2/FASLG/ATF4/NDUFAB1/LOC100524873/CYTB/SOCS3/RAC1 |
| ssc00190 | Oxidative phosphorylation | 2.1411E-05 | 73 | NDUFB7/NDUFA11/NDUFA10/LOC100522725/LOC100624067/COX7A1/NDUFS8/NDUFV1/NDUFS3/NDUFB11/ATP6V0C/NDUFS7/COX5B/NDUFV3/NDUFA3/ND3/NDUFA13/NDUFB9/COX6B/NDUFA8/ATP6V1G1/ATP5D/NDUFC2/ATP6V1A/LOC100525869/ATP5G1/NDUFB10/NDUFA2/NDUFB2/LOC100516480/LOC100156879/NDUFA7/ATP6V0D1/COX17/ATP5G2/LOC100524613/NDUFS6/COX3/COX7C/NDUFA4/NDUFB4/UQCR10/ATP6V1C2/LHPP/COX6C/ATP6V0A2/ATP6V0B/NDUFA6/NDUFB8/ND1/ATP6V1F/NDUFS5/ATP6V0E1/ATP5J/ATP5I/ATP5E/TCIRG1/COX6A1/NDUFB1/ATP8/ATP5G3/LOC100156967/NDUFV2/SDHA/NDUFS1/ATP6/NDUFA1/LOC110257420/LOC100524308/NDUFAB1/LOC100524873/CYTB/COX10 |
| ssc05016 | Huntington disease | 3.07706E-05 | 97 | NDUFB7/NDUFA11/NDUFA10/POLR2L/LOC100522725/LOC100624067/POLR2F/COX7A1/NDUFS8/NDUFV1/NDUFS3/POLR2I/NDUFB11/POLR2J/NDUFS7/GNAQ/COX5B/NDUFV3/EP300/NDUFA3/NDUFA13/NDUFB9/CLTB/COX6B/NDUFA8/ATP5D/NDUFC2/CASP8/LOC100525869/ATP5G1/NDUFB10/NDUFA2/NDUFB2/LOC100516480/LOC100156879/DNAL4/NDUFA7/CREB3L3/AP2S1/ATP5G2/SP1/LOC100524613/NDUFS6/COX3/SLC25A6/CASP9/COX7C/NDUFA4/POLR2E/NDUFB4/UQCR10/APAF1/COX6C/CREBBP/PPARGC1A/TBPL1/NDUFA6/CREB3/NDUFB8/NDUFS5/DNAL1/ATP5J/ITPR1/ATP5E/CLTC/HDAC1/CREB3L4/CLTA/COX6A1/CREB3L2/NDUFB1/CASP3/ATP8/HIP1/ATP5G3/DNAH10/SIN3A/LOC100156967/NDUFV2/SDHA/NDUFS1/POLR2G/ATP6/NRF1/NDUFA1/LOC110257420/TFAM/DCTN1/LOC100524308/PPIF/CREB3L1/NDUFAB1/LOC100524873/CYTB/DCTN2/DNAH11/VDAC1 |
| ssc05012 | Parkinson disease | 5.5072E-05 | 75 | NDUFB7/NDUFA11/NDUFA10/LOC100522725/LOC100624067/COX7A1/NDUFS8/NDUFV1/NDUFS3/NDUFB11/NDUFS7/COX5B/NDUFV3/NDUFA3/ND3/NDUFA13/NDUFB9/COX6B/NDUFA8/ATP5D/NDUFC2/LOC100525869/ATP5G1/UBE2L3/NDUFB10/NDUFA2/NDUFB2/LOC100516480/LOC100156879/UBB/NDUFA7/PARK7/ATP5G2/LOC100524613/NDUFS6/COX3/UBE2J2/SLC25A6/CASP9/COX7C/NDUFA4/NDUFB4/UQCR10/UCHL1/APAF1/COX6C/NDUFA6/SLC18A1/NDUFB8/ND1/PRKACA/NDUFS5/ATP5J/ATP5E/COX6A1/UBA1/NDUFB1/CASP3/LRRK2/ATP8/ATP5G3/SEPT5/LOC100156967/NDUFV2/SDHA/NDUFS1/ATP6/NDUFA1/LOC110257420/LOC100524308/PPIF/NDUFAB1/LOC100524873/CYTB/VDAC1 |
| ssc04714 | Thermogenesis | 9.18904E-05 | 114 | NDUFB7/NDUFA11/ADCY7/NDUFA10/LOC100522725/NDUFAF3/LOC100624067/LOC100516527/COX7A1/NDUFS8/NDUFV1/NDUFS3/NDUFB11/NDUFS7/COX5B/NDUFV3/MLST8/LOC100518575/NDUFA3/ND3/NDUFA13/PNPLA2/NDUFB9/PRKAA1/COX6B/AKT1S1/NDUFA8/HRAS/ATP5D/MAPK14/NDUFC2/LOC100525869/ATP5G1/MAP2K3/NDUFB10/NDUFA2/SMARCB1/ZNF516/NDUFB2/LOC100516480/LOC100156879/NDUFA7/CREB3L3/COX17/ATP5G2/GNAS/LOC100524613/NDUFS6/COX3/COX7C/NDUFA4/KDM3B/ARID1B/MAPK12/NDUFB4/UQCR10/COX6C/RPS6KB2/PPARGC1A/NDUFA6/ARID1A/RPS6/KRAS/CREB3/NDUFB8/ND1/PRKACA/NDUFS5/NDUFAF1/ATP5J/ATP5I/ACTL6B/NRAS/ATP5E/CREB3L4/ADCY4/COX6A1/CREB3L2/NDUFB1/ATP8/SMARCE1/ATP5G3/FRS2/LOC100156967/NDUFV2/SDHA/PRKAG2/NDUFS1/DPF3/PRKAA2/ATP6/NDUFA1/LOC110257420/SMARCD3/SOS2/CPT1B/LOC100525232/LOC100524308/NDUFAF4/RPTOR/CREB3L1/KDM3A/NDUFAB1/PLIN1/ACSL4/LOC100524873/CYTB/TSC1/COX10/BMP8B/NDUFAF2/SMARCD1/LOC100524554/SIRT6 |
| ssc04620 | Toll-like receptor signaling pathway | 0.001093375 | 49 | CD14/TLR4/TLR3/CXCL9/FADD/TLR8/LOC100515857/MAP2K2/STAT1/MAPK14/CASP8/MAP2K3/MAPK3/TOLLIP/PIK3R2/CHUK/MAP3K7/MAPK12/MAP2K6/AKT2/LBP/IFNAR1/CCL5/PIK3CD/MAPK9/NFKB1/IRF3/MAP3K8/CD86/TICAM1/IKBKG/MAP2K7/TLR6/TLR7/TAB1/IRAK1/IFNAR2/CXCL10/IL1B/SPP1/MAP2K1/MAPK1/TLR1/TBK1/IRAK4/TLR9/TAB2/TRAF6/RAC1 |
| ssc04621 | NOD-like receptor signaling pathway | 0.001204405 | 72 | P2RX7/NFKBIB/MEFV/TLR4/TYK2/FADD/TRPM2/TXN2/GABARAP/STAT1/MAPK14/CASP8/TXNIP/TNFAIP3/SHARPIN/MAPK3/NLRX1/RNASEL/GP91-PHOX/NAMPT/TP53BP1/CHUK/MAP3K7/MAPK12/CASP1/MCU/IFNAR1/LOC100155195/JAK1/CCL5/MAPK9/LOC100522011/ITPR1/NEK7/NFKB1/CTSB/TRAF2/RBCK1/IRF3/ITPR2/CARD9/CYBA/TICAM1/IKBKG/GSDMD/TAB1/BIRC3/GBP1/IFNAR2/LOC100523668/IL1B/CARD6/MAPK1/TBK1/LOC100622859/LOC100156073/MFN2/BCL2/LOC100523492/RIPK3/BCL2L1/DHX33/IRAK4/TRPM7/NLRP3/GBP2/LOC100522887/TAB2/TRAF6/XIAP/ATG12/VDAC1 |
| ssc01230 | Biosynthesis of amino acids | 0.001232517 | 39 | IDH3G/GAPDH/TALDO1/MAT2A/ACY1/SHMT2/IDH3A/PYCR3/ALDOA/PC/MAT1A/PFKL/IDH2/ENO3/MTR/GPT2/LOC110255953/RPE/ENO1/ASL/GPT/PRPS1/ACO2/CBS/BCAT1/ALDOB/TKT/ALDH18A1/CPS1/PYCR2/RPIA/GOT1/SDSL/ARG1/TAT/TPI1/PAH/PFKM/BCAT2 |
| ssc00770 | Pantothenate and CoA biosynthesis | 0.002016731 | 14 | PPCDC/DPYD/COASY/BCAT1/ENPP3/DPYS/PANK2/AASDHPPT/ENPP1/VNN2/UPB1/VNN3/BCAT2/VNN1 |
| ssc03018 | RNA degradation | 0.002434839 | 40 | LSM4/EXOSC4/LSM7/SKIV2L/DCPS/WDR61/EXOSC7/LSM2/CNOT7/DIS3L/PFKL/PAN3/CNOT1/ENO3/DCP1A/DCP2/PAN2/EXOSC6/DDX6/ENO1/EXOSC5/PAPD5/EXOSC2/CNOT8/TOB1/XRN1/LSM5/HSPD1/DHX36/NUDT16/CNOT6L/PABPC1/DIS3/CNOT3/BTG2/EDC3/PFKM/PNPT1/TOB2/CNOT6 |
| ssc04370 | VEGF signaling pathway | 0.002585758 | 30 | RAC3/HSPB1/MAP2K2/HRAS/MAPK14/MAPK3/CDC42/PRKCA/KDR/PIK3R2/CASP9/PRKCB/PPP3CB/MAPK12/PLA2G4B/AKT2/PPP3R1/KRAS/RAF1/PIK3CD/NRAS/BAD/PLA2G4A/VEGFA/MAPKAPK2/MAP2K1/MAPK1/SPHK2/RAC1/PLCG1 |
| ssc04140 | Autophagy - animal | 0.003240138 | 64 | RUBCN/MLST8/BNIP3/PRKAA1/DAPK1/MAP2K2/ULK2/AKT1S1/HRAS/GABARAP/ATG101/LAMP2/VAMP8/MAPK3/CFLAR/PDPK1/ATG14/PIK3R2/PTEN/IGF1R/RB1CC1/MAP3K7/WIPI2/HIF1A/UVRAG/CAMKK2/RPS6KB2/AKT2/KRAS/RAF1/RAB7A/LAMP1/EIF2AK4/PRKACA/MTMR4/PIK3CD/MAPK9/ITPR1/CTSB/NRAS/BAD/ATG9A/RRAS/PIK3C3/ATG4B/PRKAA2/MAP2K1/MAPK1/SH3GLB1/HMGB1/ATG10/BCL2/BCL2L1/BECN1/ATG2B/RPTOR/MTMR14/IGBP1/ATG9B/ATG4C/TSC1/TRAF6/RAB7B/ATG12 |
| ssc01524 | Platinum drug resistance | 0.003793761 | 38 | ERCC1/LOC100151901/FADD/POLH/-/CASP8/MAPK3/SLC31A1/PDPK1/PIK3R2/LOC100739163/CASP9/MDM2/APAF1/AKT2/PIK3CD/BID/BAD/MGST2/CDKN1A/MGST3/CASP3/MSH6/BIRC3/ATM/FAS/TOP2A/GSTO1/MSH3/LOC100739508/MAPK1/REV3L/LOC100622859/BCL2/BCL2L1/FASLG/XPA/XIAP |
| ssc00604 | Glycosphingolipid biosynthesis - ganglio series | 0.006734012 | 11 | B3GALT4/HEXB/ST8SIA5/ST3GAL5/GLB1/ST6GALNAC3/HEXA/ST6GALNAC4/ST6GALNAC6/ST3GAL2/ST6GALNAC5 |
| ssc04142 | Lysosome | 0.009454037 | 58 | CTSC/ATP6V0C/SMPD1/GNS/CLN5/IDUA/CLTB/LAMP2/ASAH1/CTSW/AP4S1/HEXB/ENTPD4/ATP6V0D1/GNPTAB/AP1G1/MANBA/DNASE2B/ARSB/SGSH/GAA/ATP6V0A2/ATP6V0B/LAMP1/CTSZ/ACP2/LAPTM4A/AP1M1/GLB1/PPT1/NAGPA/GM2A/SUMF1/CD164/CTSB/AP4M1/CTSO/CTSH/TCIRG1/SLC17A5/CLTC/SCARB2/CLTA/CTSF/GLA/AP3S2/MFSD8/HEXA/NPC1/ARSG/AP1S1/AP3M2/GALC/CTNS/GGA3/NAGA/NEU1/PSAP |
| ssc04137 | Mitophagy - animal | 0.013065663 | 33 | FIS1/BNIP3/CALCOCO2/CSNK2B/HRAS/GABARAP/UBB/TBC1D17/SP1/TFEB/NBR1/HIF1A/KRAS/RAB7A/USP8/MAPK9/NRAS/CSNK2A1/MITF/ATG9A/RRAS/TBC1D15/FUNDC1/CSNK2A2/TBK1/CITED2/RHOT1/MFN2/BCL2L1/BECN1/ATF4/ATG9B/RAB7B |
| ssc00051 | Fructose and mannose metabolism | 0.013522926 | 19 | TSTA3/GMPPA/ALDOA/PFKL/KHK/PMM1/GMDS/TIGAR/ALDOB/FBP1/ENOSF1/MPI/SORD/HKDC1/TPI1/PMM2/PFKM/TKFC/PFKFB1 |
| ssc05161 | Hepatitis B | 0.013911227 | 71 | TLR4/EP300/TLR3/TYK2/NFATC3/FADD/TGFBR2/MAP2K2/HRAS/STAT1/MAPK14/CASP8/MAP2K3/MAPK3/ARAF/CREB3L3/PRKCA/PIK3R2/CASP9/PRKCB/CHUK/DDX3X/MAP3K7/MAPK12/JAK2/MAP2K6/IFIH1/APAF1/CREBBP/CCNA2/AKT2/KRAS/RAF1/CREB3/IFNAR1/JAK1/PIK3CD/MAPK9/BRAF/YWHAZ/NFKB1/NRAS/BID/IRF3/BAD/CDKN1A/TICAM1/CREB3L4/IKBKG/MAP2K7/CREB3L2/CDK2/CASP3/TAB1/IRAK1/FAS/MAP2K1/MAPK1/SOS2/TBK1/CASP10/SLC10A1/BCL2/IRAK4/CREB3L1/FASLG/ATF4/TAB2/ELK1/TRAF6/HSPG2 |
| ssc00270 | Cysteine and methionine metabolism | 0.015097224 | 25 | TST/MAT2A/ADI1/MAT1A/MPST/LDHA/MTR/MRI1/AHCY/GCLC/SRM/AHCYL2/CBS/BCAT1/GSS/GOT1/SDSL/AHCYL1/TAT/MTAP/KYAT3/DNMT3B/DNMT3A/BCAT2/MDH2 |
| ssc00600 | Sphingolipid metabolism | 0.021170698 | 25 | CERS4/SMPD1/SPTLC2/ASAH1/GAL3ST1/SMPD2/DEGS2/PLPP1/GLB1/UGT8/ACER3/SGPP2/CERS5/SGPL1/PLPP2/SMPD4/GLA/SPTLC1/B4GALT6/SMPD3/GALC/ASAH2/CERS6/SPHK2/NEU1 |
| ssc05140 | Leishmaniasis | 0.022971225 | 33 | NFKBIB/TLR4/STAT1/MAPK14/MAPK3/GP91-PHOX/SLA-DQB1/PRKCB/MAP3K7/MAPK12/SLA-DRB1/ITGB1/JAK2/SLA-DOA/JAK1/NFKB1/ITGA4/CYBA/IFNGR1/C3/IFNGR2/TAB1/IRAK1/IL1B/NCF2/MAPK1/IRAK4/ITGAM/MARCKSL1/TAB2/ELK1/TRAF6/NCF4 |
| ssc05152 | Tuberculosis | 0.023442267 | 71 | CD14/NFYA/ATP6V0C/MRC1/TLR4/EP300/FADD/STAT1/MAPK14/LAMP2/CASP8/MAPK3/CD74/ATP6V0D1/RAB5B/ARHGEF12/CASP9/SLA-DQB1/PPP3CB/FCGR2B/MAPK12/LSP1/SLA-DRB1/JAK2/RFX5/CAMK2D/APAF1/CREBBP/ATP6V0A2/ATP6V0B/AKT2/PPP3R1/RAF1/RAB7A/LAMP1/LBP/SLA-DOA/JAK1/NFYC/MAPK9/NFKB1/BID/BAD/RFXANK/CARD9/TCIRG1/IFNGR1/IRAK2/TLR6/C3/IFNGR2/CASP3/IL10RA/IRAK1/FCER1G/CEBPB/PIK3C3/IL1B/HSPD1/MAPK1/TLR1/CASP10/BCL2/PLA2R1/CORO1A/IRAK4/TLR9/ITGAM/TRAF6/SPHK2/CAMK2G |
| ssc03050 | Proteasome | 0.023830579 | 23 | PSMC3/PSMB7/PSMB3/PSMA7/PSMB4/PSMB1/PSMD13/PSMC5/PSMB6/LOC102157770/PSMB5/PSMD4/PSME1/PSMF1/PSMD8/PSMD3/PSMC4/PSME2/PSMA4/PSMB2/PSMB10/PSMD6/PSMC6 |
| ssc04120 | Ubiquitin mediated proteolysis | 0.024174995 | 62 | ELOB/ANAPC11/UBE4A/STUB1/UBE2D3/UBR5/CDC34/TRIP12/UBE2L3/HERC1/UBE2M/UBE2H/CDC27/CBL/NEDD4/RHOBTB1/UBE2J2/MDM2/SYVN1/HERC2/FZR1/SKP2/HERC4/FBXW11/ANAPC4/PIAS1/UBE2Q2/CDC23/UBE2O/ANAPC1/TRIM37/ANAPC2/BIRC6/PPIL2/SMURF2/UBE4B/UBA6/UBA1/UBE2I/CBLB/BTRC/UBE2D2/UBE2S/KLHL9/KEAP1/BIRC3/PIAS2/KLHL13/HERC3/UBE2R2/LOC100622859/UBE2D4/CUL4B/UBE2F/HUWE1/NEDD4L/ELOC/UBE3A/TRAF6/SOCS3/MGRN1/XIAP |
| ssc00520 | Amino sugar and nucleotide sugar metabolism | 0.027203385 | 26 | TSTA3/PGM1/NPL/GALT/GALE/GMPPA/LOC100524254/HEXB/AMDHD2/GALK1/UGDH/PMM1/GMDS/CYB5R1/UGP2/RENBP/UAP1L1/NANS/MPI/GPI/HEXA/HKDC1/LOC100622386/GNE/PMM2/CMAS |
| ssc04622 | RIG-I-like receptor signaling pathway | 0.029811957 | 29 | NFKBIB/FADD/MAPK14/CASP8/NLRX1/CHUK/ISG15/DDX3X/MAP3K7/MAPK12/CYLD/IFIH1/MAPK9/NFKB1/SIKE1/TRAF2/IRF3/TRIM25/TBKBP1/PIN1/IKBKG/AZI2/CXCL10/TBK1/CASP10/TRAF6/TKFC/ATG12/RNF125 |
| ssc04066 | HIF-1 signaling pathway | 0.033120232 | 45 | TFRC/ELOB/TLR4/EIF4EBP1/ARNT/EP300/GAPDH/MAP2K2/MAPK3/TIMP1/LTBR/GP91-PHOX/TEK/PRKCA/PIK3R2/IGF1R/PRKCB/PDHA1/HIF1A/IL6R/ENO3/CAMK2D/PDK1/CREBBP/RPS6KB2/AKT2/RPS6/PIK3CD/NFKB1/ENO1/CDKN1A/CDKN1B/ANGPT4/IFNGR1/EGLN3/IFNGR2/VEGFA/MAP2K1/MAPK1/HKDC1/BCL2/IGF1/ELOC/CAMK2G/PLCG1 |
| ssc04215 | Apoptosis - multiple species | 0.03349846 | 17 | FADD/CASP8/SEPT4/CASP9/BOK/APAF1/MAPK9/NGFR/BID/BIRC6/CASP3/BIRC3/LOC100622859/BCL2/BCL2L1/BECN1/XIAP |
| ssc04210 | Apoptosis | 0.042603655 | 61 | CTSC/TUBA8/FADD/MAP2K2/HRAS/TUBA4A/CASP8/MCL1/MAPK3/TNFSF10/CTSW/CFLAR/SEPT4/ENDOG/LMNA/PDPK1/LOC100158003/PIK3R2/CASP9/LOC100624226/CHUK/CSF2RB/APAF1/AKT2/KRAS/RAF1/CTSZ/PARP4/PIK3CD/NGF/MAPK9/ITPR1/NFKB1/CASP6/CTSB/NRAS/TRAF2/BID/BAD/ITPR2/CTSO/CTSH/DDIT3/IKBKG/CASP3/BIRC3/ATM/CTSF/FAS/MAP2K1/MAPK1/LOC100622859/CASP10/PIDD1/BCL2/BCL2L1/FASLG/ATF4/XIAP/DAXX/LOC100127131 |
| ssc04217 | Necroptosis | 0.042603655 | 61 | LOC100625850/CHMP2A/SMPD1/FTL/TLR4/TLR3/TYK2/CHMP6/FADD/CHMP1B/STAT1/CASP8/TNFAIP3/SHARPIN/TNFSF10/CFLAR/GP91-PHOX/SLC25A6/LOC100624226/PPIA/CASP1/JAK2/CYLD/CAMK2D/PLA2G4B/IFNAR1/PARP4/JAK1/MAPK9/LOC100522011/VPS4A/TRAF2/CHMP2B/RBCK1/BID/MLKL/LOC100512448/TICAM1/FTH1/IFNGR1/IFNGR2/PLA2G4A/BIRC3/FAS/IFNAR2/VPS4B/CHMP4A/IL1B/LOC100622859/HMGB1/BCL2/RIPK3/CHMP4B/CHMP1A/TRPM7/NLRP3/FASLG/SPATA2/XIAP/VDAC1/CAMK2G |
| ssc05014 | Amyotrophic lateral sclerosis (ALS) | 0.044351169 | 23 | CCS/PRPH/MAPK14/MAP2K3/ALS2/CASP9/PPP3CB/MAPK12/CASP1/DERL1/MAP2K6/APAF1/PPP3R1/SLC1A2/BID/BAD/CASP3/TOMM40L/BCL2/BCL2L1/TOMM40/DAXX/RAC1 |
